# Algorithm Perception When Using Threat Intelligence in Vulnerability Risk Assessment

**DOI:** 10.1111/risa.70178

**Published:** 2026-01-30

**Authors:** Sarah van Gerwen, Aurora Papotti, Katja Tuma, Fabio Massacci

**Affiliations:** ^1^ Informatica Vrije Universiteit Amsterdam The Netherlands; ^2^ Computer Science TU Eindhoven Eindhoven The Netherlands; ^3^ DISI University of Trento Trento Italy

**Keywords:** algorithm aversion, experiment, threat intelligence, vulnerability risk assessment

## Abstract

Recent government and commercial initiatives have pushed for the use of the automated, artificial intelligence (AI)–based, analysis of cyber threat intelligence. The potential bias that might be present when evaluating threat intelligence coming from human and AI sources has to be better understood before deploying automated solutions to production. We present a controlled experiment with n=57 master students who had a mix of experience in security and machine learning to measure the bias introduced by the source of intelligence (human vs. AI). Each participant analyzed eight threat intelligence reports from the Dutch National Cyber Security Center where the source of the final recommendation was manipulated as for coming from a human expert or an AI algorithm. Our findings revealed that participants tended to disagree with the recommendation when it was coming from AI. While expertise on ML did not have any impact, we found that participants with more security expertise tended to agree with the recommendation. In contrast, we found that the perceives bias was statistically equivalent (TOST) whether the recommendation was coming from a human or from an AI. The only (expected) factor which had an impact on perceived bias was when participants disagreed with the recommendation (irrespective whether it was human or AI). These results provide insight on the possible impact of introduction on AI on rank‐and‐file Tier 1 SOC analysts. The generalization of our results to professional practice requires more experiments with experienced security professionals.

## 1 Introduction

US Federal executive orders push federal agencies and private companies to “streamline access to cybersecurity data to drive analytics for identifying and managing cybersecurity risks” (Biden 2021). To do so, organization needs reliable and manageable information on impact and likelihood, which are the two basic pillars of risk analysis.

While measures to understand cybersecurity impact are well understood and standardized, estimating likelihood has always been a hard problem (Bozorgi et al. 2010, Allodi and Massacci 2014). Let alone capturing adversaries and game theoretic aspects (Hausken et al. 2024), likelihood estimation requires processing a significant amount of data (Allodi and Massacci 2017), and organizations are increasingly overwhelmed by the amount of threat intelligence data (Bouwman et al. 2020, de Smale et al. 2023).

The traditional solution to the problem has been relying on the national security agencies for prioritization. For example, the Dutch National Cyber Security Centre (NCSC) bases the risk assessment of the likelihood of vulnerability exploitation on a combination of technical knowledge of the vulnerability and threat intelligence. It is essentially an ad hoc scoring systems for likelihood calculation with all the known limitation of risk matrices (Cox 2008). The “algorithm” is provided but eventually the likelihood value is determined by two/three fields based on expert judgment (e.g., “Likely to be exploited in the near future”). Italy and China do not even report the individual metrics, just the global risk level. In a nutshell, *the cyber risk analyst has to trust the national security expert*. Experiments have shown that intelligence expert judgment may not be reliable (Irwin and Mandel 2023) and that participants may consider the suggestions by experts biased (Commons et al. 2004,2012).

Artificial intelligence (AI) and machine learning (ML) seem an algorithmic alternative to blindly trusting the national experts. In other domains, such as predicting the risk of extreme weather events, models are openly published and discussed (Bellprat et al. 2019).

A recent industry initiative is the Exploit Prediction Scoring System (EPSS) with over 200 members including among others the US Department of Homeland Security and the Cybersecurity and Infrastructure Security Agency. EPSS gathers threat intelligence on the web and tries to predict potential exploits using ML (Jacobs et al. 2023). One of the key aspects is that the prediction of the likelihood is completely back‐box. A paper describes the model at a very high level (Jacobs et al. 2023) and the company producing the EPSS scores, only makes available the final prediction. Essentially, *the cyber risk analyst has to trust the AI algorithm*. Also in this case, several experiments have shown the presence of algorithmic bias in which participants with different level of domain knowledge have shown both algorithm aversion (Dietvorst et al. 2015, Castelo et al. 2019) and algorithm appreciation (Logg et al. 2019, Rieger et al. 2023). Such change in sign of appreciation depends often on the actual knowledge: either in the algorithmic knowledge or the domain knowledge.

At this point, an interesting scenario present itself: *in presence of such uncertain, essentially black‐box, threat intelligence recommendations by the AI or the human what would cyber risk analysts do?* Would they exhibit algorithmic risk aversion or algorithm appreciation? Would such bias change depending on their expertise (e.g., security knowledge vs. ML knowledge) as the literature seems to suggest?

The contribution of this paper is to report the result of a controlled experiment to answer the questions above by using threat intelligence report as close as possible to the ones actually used on the field. We adopted a Taguchi Balanced design (Tsui 1992) as made available from Massacci et al. (2024) with a total of n=57 master students in computer science with a mix of background in security and ML. We tasked our participants with assessing English‐translated threat intelligence reports from the Dutch NCSC. Due to the narrow student sample and constrained laboratory setting of the current experiment, the current study should be interpreted as a preliminary effort, a plausibility probe, to explore whether this phenomenon merits further investigation in more realistic settings. In addition, choosing students as the population can lead to limitations such as replicating realistic challenges such as decision‐making pressures, contextual familiarity, or accountability constraints. We think that training the participants in advance may reduce the severity of this issue, but it cannot eliminate the gap in real‐world experience; therefore, the findings of this paper to be considered generalized, require further experiments including cybersecurity professionals.

Our findings revealed that participants tended to disagree with the recommendation when it was coming from AI. While expertise on ML did not have any impact, we found that participants with more security expertise tended to disagree less with the recommendation. However, the source had no impact on the perceived bias, and intuitively, only when the participants disagreed they found the recommendation to be biased. The results show that reporting data from AI might be trusted slightly less than human recommendation albeit not to the point of being seen as explicitly biased.

Most importantly, the perceived bias by AI and human sources was found to be statistically equivalent using two one‐sided tests. This result is particularly important because it counters simplistic narratives about widespread algorithm aversion. That AI and human sources were judged similarly in bias perception (even in an unfamiliar and uncertain domain) is a valuable finding.

Such findings are useful to assess the likely reactions by junior cyber risk analysts who are normally the rank‐and‐file officers of cyber‐security operations centers (SOCs) as Tier 1 analysts (Vielberth et al. 2020).

The generalization of our results to professional practice requires more experiments with security professionals (e.g., SOC Tier 3 analysts or threat intelligence specialists) as they might have different frames of mind both in general (Kahneman and Klein 2009) and specifically for security risk analysis (Labunets et al. 2023).

In the rest of the paper, we introduce some terminology (Section 2) and discuss the theoretical background and some key gaps in the literature (Section 3). Then we introduce our research questions (Section 4) and briefly summarize the analysis procedure (Section 5). Section 7 describes the experimental artifacts, whereas Section 8 summarizes the experimental design. Ethical aspects are summarized in Section 6. Eventually, the results of the experiment are presented (Section 10) and discussed with their potential implications (Section 12 and the threats to validity (Section 11). Section 13 concludes the paper.

### 1.1 Artifact Availability Statement

The data that support the findings of this study are openly available in Zenodo at https://doi.org/10.5281/zenodo.16418849 (van Gerwen et al. 2025).

## 2 Terminology

In this section, we present some basic terminology of the artifact and topic of analysis used in this paper.

*Software vulnerabilities* can be defined as flaws in the design, implementation, or configuration of software systems. The exploitation of these flaws violate the security and/or safety of the system. Vulnerability risk assessment is the process of identifying, prioritizing, and mitigating these weaknesses. Risk assessment of vulnerabilities dictates prioritization and ultimately, further mitigation steps (Initiative 2012).

*Threat intelligence* is evidence‐based knowledge representing threats that can inform decisions (Tounsi and Rais 2018). This evidence‐based knowledge can be seen as the result of a process that has been described as sensemaking (Klein et al. 2007). Sensemaking can be seen as a form of abductive reasoning where the most plausible conclusion about a situation is the end goal (Klein et al. 2023). Threat intelligence reports can be technical or narrative and vary between a multitude of standardized and non‐standardized formats (Yang and Lam 2020, Irshad and Basit Siddiqui 2023). Ontologies exist for the standardization of cyber threat intelligence (Tounsi and Rais 2018). Two well‐known vocabularies are STIX (Structured Threat Information eXpression) (Committee 2023) and MITRE ATT&CK (Corporation 2023). Domain objects and relationship objects are part of the structured language STIX. Adversary tactics and real‐world techniques are part of the vocabulary of MITRE ATT&CK. Uncertainty information is not covered by these ontologies. Perry et al. (2019) mention that free‐text reports do often include similar attack details such as the attacked party, related attacks, and a technical analysis.

To date, an assessment of the risk behind the threat of exploitation of a software vulnerability is often a manual process (Khan and Parkinson 2018). The analysis of advanced persistent threats by Di Tizio et al. (2022) had to sieve through over 150 threat intelligence reports written by human experts. What is therefore distilled as actionable (“high or critical”) threat intelligence is a product of human decision‐making. In other words, in most if not all cases there remains a human in the loop. The recent research and practice has been pushing toward the automation of threat intelligence collection and analysis by means of AI models as a “black‐box” such as the mentioned EPSS industry initiative (Jacobs et al. 2023).

## 3 Theoretical Background and Related Work

### 3.1 Risk Communication, Source Credibility, and Trust

A considerable amount of literature has been published on risk and uncertainty communication in the past (Balog‐Way et al. 2020, Spiegelhalter 2017). This literature spans multiple fields, including threat intelligence (Mandel 2020), and focuses on different aspects of communication. In their overview, Balog‐Way et al. (2020) put forward three areas that underline risk communication research: messengers, message attributes, and audiences.

Research with respect to *messengers* often focuses on source credibility and trust (Balog‐Way et al. 2020). Research in source credibility illuminates how different aspects of a source influence the extent to which a receiver senses this source as reliable and in turn, accepts and uses the source's message (Hovland and Weiss 1951). Within the field of cyber threat intelligence, past research focused on user evaluations of sources to detect deception (Ormond et al. 2016) as well as CERT experts' credibility evaluation of messages on Twitter (Basyurt et al. 2022). Trustworthiness and expertise have been identified as key aspects of source credibility (Wiener and Mowen 1986, Flanagin and Metzger 2020). The trustworthiness of a source is explained as the perception that a source is objective and honest while expertise is explained as the perception that a source has obtained the required skills and knowledge for the task at hand (Hovland and Weiss 1951). Previous research indeed suggests that the perceived usefulness of a message and the adoption of the message is influenced by source credibility in general or specifically by source expertise and source trustworthiness in many fields such as electronic worth of mouth (Ismagilova et al. 2020) and online reviews (Aghakhani et al. 2022).

However, the aforementioned research mainly focuses on how the broader public is influenced by different sources that are assumed to be human. In the field of threat intelligence, it is often experts who are tasked with making credibility assessments. Moreover, the concept of trustworthiness of sources in threat intelligence is complicated, since the exact data often cannot be shared, and the receiver of the risk communication must therefore rely on a multitude of different institutions, individuals, and systems. Some of these sources might be known to the receiver, but many are unknown. Additionally, some of these sources are possibly not human or the messages attached to these sources are partly based on algorithmic decisions. Furthermore, besides possible misinformation, it is also possible that sources are intentionally spreading disinformation. There is therefore a need to understand whether there is a difference between human and algorithmic sources within the specific context of threat intelligence. Additionally, Wallace et al. (2020) showed that perceived bias influenced source credibility beyond effects of trustworthiness, expertise, and likeability. Therefore, trustworthiness and bias should be differentiated when it comes to source credibility.

Besides source credibility, trust is a major area of study when it comes to messengers (Balog‐Way et al. 2020). Following the conceptualization of Gill et al. (2024), trust is seen as organizational trust which is composed of trust attitudes (affect‐based and cognition‐based) and trust intentions (reliance and disclosure). Affect‐based trust attitudes are relational and grounded in an emotional bond, whereas cognition‐based trust is more rational and focused on the perception of reliability and competence (Gill et al. 2024). Reliance is about depending on the source in question, whereas disclosure is about the inclination to share sensitive information (Gill et al. 2024). The current research can be framed as focusing on the trust intention of reliance. It is out of the scope of the current study to measure the difference between affect‐based trust and cognition‐based trust.

Research with respect to *message attributes* often focuses on framing, affects and uncertainty communication (Balog‐Way et al. 2020). Communicating uncertainty successfully with respect to threat intelligence has been object of inquiry for a long time (Mandel 2020). This is the case because there is an effect of the way uncertainty is represented on decision‐making (Durbach and Stewart 2011). Although transparency about uncertainty is needed, it can also lead to less trust and less effective decision‐making (Balog‐Way et al. 2020). Moreover, when introducing AI as part of the equation, information about the accompanied uncertainty can be insufficient or altogether unavailable. At the same time, utility of threat intelligence is dependent on the quality of the intelligence which in turn, is contingent on a judgment of how uncertain or biased information is (Wagner et al. 2019).

Research on *audiences* focuses often on different aspects of the audiences risk perception and how communication affects these perceptions (Balog‐Way et al. 2020). Risk perceptions and risk based decision‐making are discussed in more detail in the Section 3.2.

Different theories have been posited and researched to incorporate and situate these different areas of risk communication. One such theory is the risk‐as‐feeling theory, which suggests that messages do not only have a cognitive component but that their persuasiveness is strongly influenced by affective components (Loewenstein et al. 2001). Closely related is research concerning the affect heuristic (Slovic et al. 2007, Finucane et al. 2000) that posits that mental representations of an object or event are constructed with varying affect degrees. These degrees of affect are used as a cue for judgment (Slovic et al. 2007). Although most research on the affect heuristic is performed outside of the current field, there is prior research in cybersecurity that tested the affect heuristic (van Schaik et al. 2020) found that parts of the conceptualization of affect (i.e., valence) indeed impacted the risk perception of participants in the field of cybersecurity. To avoid bias in one direction or another, it might be important to present the machine or the human decision as neutrally or minimally as possible.

Another theory is the social amplification of risk framework (Kasperson et al. 2022). The idea behind this theory is that the communication of the risk of hazards can amplify or attenuate the risk itself (Kasperson et al. 2022). This is the case because technical issues that will interact with the specific hazard can arise from public perceptions, responses, and behaviors (Kasperson et al. 2022). Audiences are therefore active participants in the amplification or attenuation of risks. Prior research with respect to threat intelligence shows, for example, that cybersecurity firms focus their threat reporting on major firms instead of smaller firms and civil society (Maschmeyer et al. 2021, Egloff 2020). This underrepresentation can steer academic and policy debates away from these underreported threats which can amplify the risk itself. Interestingly, recently Makridis et al. (2024) argued that media coverage of cyber conflicts is driven by different indicators than non‐cyber threats. Mainly, Makridis et al. (2024) found that coverage was heightened for cyber operations that included technical novelty such as zero‐day exploits.

**Key Gap**. Most existing studies on risk assume that the source is human, overlooking the increasing relevance of nonhuman, algorithmic sources‐especially in the context of cyber threat intelligence. Furthermore, while source credibility research has extensively explored trustworthiness and expertise, it often neglects to differentiate between trust and perceived bias. Additionally, most research focuses on general public audiences, whereas in cyber threat intelligence, expert users must assess credibility without direct access to source data, relying instead on a fragmented web of unknown sources. Therefore, there is need to investigate how human versus algorithmic sources are perceived and trusted in expert cybersecurity contexts.

### 3.2 Risk‐Based Decision‐Making

In this section, we discuss the theoretical background on risk‐based decision‐making under uncertainty and the role of algorithmic bias in such decisions and how it impacts the potential implications of using AI for threat intelligence.

*Risk Decisions Under Uncertainty and Time Pressure*. In presence of time pressure and incomplete information, risk decision makers need to make certain trade‐offs. Risk analysis research has been interested in understanding risk perception (Slovic 1987) and risk decisions under uncertainty and extreme time pressure. Early work by Kahneman and Tversky (1979) explores heuristics and biases which laid out the foundations for cognitive limitations in risk decisions. Subsequently, Fischhoff (1982) and Dubois (2010) place debiasing techniques in the foreground and investigate the roles of uncertainty theories. The aim of debiasing techniques, such as hindsight bias and overconfidence, is to improve decision‐making accuracy by understanding and addressing the psychological processes that lead to biased judgments. Particularly relevant in this space is also the imprecise probabilities theory in risk analysis, framing the space of risk decisions when decision makers are faced with incomplete information. Dubois (2010) argues integrating objective and subjective approaches to better capture expert judgments under such conditions. On this point, Gigerenzer and Gaissmaier (2011) review heuristic decision‐making and find that, in uncertain, real‐world problems, the literature shows that heuristics can often be more accurate than complex “rational” strategies.

Further, when risk decisions are made in a group context, such as during safety analysis with methods like Failure Mode and Effects Analysis (FMEA) (Zhang and Liu 2022, Collier et al. 2023), expert judgment plays a pivotal role in group decision‐making dynamics, as argued by Otway and von Winterfeldt (1992), Winter et al. (2022), and Sniezek (1992). Such advancements have over decades led to more robust risk analysis methods, improving decision quality in high‐stakes, time‐sensitive situations.

*Machine Augmented Risk Decisions in the Intelligence Domain*. Kelly et al. (2023) surveyed factors contributing to the acceptance of AI and found that not all papers define what AI is nor do they check participants understanding of AI, which makes result comparison difficult. While the AI Device Use Acceptance model emerged as promising theoretical model, more research is required to assess the actual uptake of AI.

Threat intelligence environments are complex situations where information is aggregated from various sources (Paté‐Cornell 2002, Menkveld 2020). Time pressure (Irwin and Mandel 2019, Florig et al. 2001, Morgan et al. 2001, Jaspersen and Montibeller 2015), secrecy of information (Pedersen and Jansen 2019), the possibility of deception (Whitesmith 2019), and the high stakes of decisions (Jensen 2012) are characteristics of these situations. In addition, the process of sharing obtained information and the data it is based upon might be limited for operational, strategic, or legal reasons (Wagner et al. 2019). Due to this highly restricted information sharing, accurate representations of risk‐based decision‐making in security communities are rare (Wagner et al. 2019, Landon‐Murray 2016). This leads to information being lost or only being available to a highly limited group of organizations and individuals within the organization. The net result is that “normal organizations” have to trust the intelligence that they receive.

Past research investigated how uncertainty is handled by human analysts in *risk decisions when augmented with machine reasoning*. Paté‐Cornell (2024) investigated the discrepancy between the risk attitudes embedded in AI decision algorithms and the preferences of actual decision makers and those affected by their decisions. According to Paté‐Cornell, this discrepancy can lead to significant issues in risk management, thus making AI factors transparent and adjustable to better align with the preferences of decision makers and stakeholders is advisable. Irwin and Mandel (2023) conduct experiments with 41 intelligence analysts to investigate their preferences in communicating uncertainty. Interestingly, while nonexperts generally prefer numeric formats, experts are divided, and both groups often conflate probability and confidence, leading to inconsistent and sometimes incoherent numeric translations of verbal probabilities. Karvetski et al. (2020) conduct an empirical evaluation of structured methods used to process intelligence data, and often promoted in intelligence organizations, and find that such methods were ineffective and even impeded some aspects of quality in probability judgments in intelligence analysis. Isaksen and McNaught (2019) conduct semi‐structured interviews with senior consumers of military intelligence and focus group interviews with groups of analysts, and found that respondents found it difficult to conceptualize uncertainty analytically. This further highlights the importance of understanding human bias toward machine‐augmented risk decisions in threat intelligence.

Previous work conducted *validation of bias in threat intelligence*. For example, Mandel and Barnes (2014) analyzed intelligence forecasts and found that under‐confidence (assigning more uncertainty to a given forecast than needed) was more pronounced in forecasts that were important for policy‐making. Menkveld (2020), analyzing a set of reports from the Dutch secret services (AIVD), observed that analysts had to fill in larger gaps and were less confident when complexity in intelligence problems increased.

Ranade et al. (2021) used human cybersecurity experts to validate that generated fake cyber threat intelligence was considered equally true as authentic threat intelligence. Whyte (2022) crafted scenarios and used military professionals as well as workshop participants to observe that, within a cyber conflict scenario, algorithm aversion was found unless there was the perception of humans in the loop. This was the case even though the information presented stayed the same. Whitesmith (2019) used a crafted scenario and a population consisting of students and staff from the university and different governmental departments. The study found that the possibility of deception is most likely to be considered in cases where information contradicts already held beliefs. Pedersen and Jansen (2019) used crafted scenarios to show that analysts gave more weight to secret information in comparison to open‐source information. Their sample consisted of a combination of intelligence studies students and intelligence professionals. However, this effect was no longer found when the problems decreased in complexity (Pedersen and Jansen 2019) or when only meta‐information was provided (Mandel et al. 2023). For the study on meta‐information, Mandel et al. (2023) used professional intelligence analysts and meta‐information pieces of information based on the NATO standard.

Isaksen and McNaught (2019) performed semi‐structured interviews with senior consumers of military intelligence and intelligence analysts. The study found that consumers and analysts perceived the analytic conceptualization of uncertainty to be difficult and suggested using a differentiated, in both level of uncertainty and situation, framework for uncertainty communication.

**Key Gap**: A large body of knowledge deals with the problem of combining expert judgments to raise the validity of the final decision (Stroop 1932, Scholz and Hansmann 2007), and algorithm aversion widely studied in machine‐augmented risk decision‐making (Burton et al. 2020, Mahmud et al. 2022, Hou and Jung 2021, Dietvorst et al. 2015, Feng and Gao 2020, Prahl and Van Swol 2017). But little focus was put on investigating whether judgments coming from experts and AI can be differently perceived as biased in the threat intelligence domain.

### 3.3 Algorithm Aversion and Appreciation

*Research on Judgment*. When looking at research on judgment, there is a difference in the influence of algorithmic judgment in comparison to human judgment on human decision‐making (Dietvorst et al. 2015, Castelo et al. 2019, Logg et al. 2019, Hou and Jung 2021). This influence is connected to the preference for using algorithmic or human conclusions (Dietvorst et al. 2015). A much debated question is the effect of the difference between algorithmic and human judgment. Studies have shown that human judgment is preferred over algorithmic judgment, even in cases where algorithms outperform humans (Dietvorst et al. 2015, Castelo et al. 2019). This phenomenon is called algorithm aversion. In contrast, multiple studies found an opposite effect coined as algorithm appreciation (Logg et al. 2019, Rieger et al. 2023). Here, findings illustrated that people prefer algorithmic judgment to human judgment.

To account for the differences, several inquiries have been conducted into the factors that constitute algorithm aversion and appreciation. Studies are numerous (Burton et al. 2020, Mahmud et al. 2022) and possible factors include corroboration with own decision (Logg et al. 2019) and quality of information provided (Gönül et al. 2006). Expertise seems to have a mitigating effect and can even induce the opposite effect (Hou and Jung 2021). In addition, within a cyber conflict scenario where algorithmic threat intelligence was used, the perception alone of humans in the loop reduces algorithm aversion, even when the judgments are the same (Whyte 2022). Without the perception of a human in the loop, experts showed algorithm aversion in this scenario (Whyte 2022). This experiment was validated in the domain of cyber conflicts.

In recent years, more investigations have been made to develop a conceptual framework or model to capture algorithm aversion. Burton et al. (2020) proposed a framework consisting of five factors: expectations and expertise, decision autonomy, incentivization, cognitive compatibility, and divergent rationalities. Mahmud et al. (2022) presented a framework consisting of four factors: high‐level factors, individual factors, task factors, and algorithm factors. These two conceptualizations can be seen as complementary and touch upon similar ideas. What can be construed from both conceptualizations is that algorithm aversion is highly dependent on the current context and population. Studies review some experiments in several domains such as the hiring process, diagnostic decision‐making, and financial forecasting but not in the field of threat intelligence.

Therefore, in the present study, the perception of algorithmic recommendations will be studied as it is pertaining to the usage of threat intelligence in likelihood risk assessment. The focus of the study is on individual decision‐making and not on the organizational and societal factors that also play a large role in algorithm perception.

**Key Gap**: Validation studies with human participants measuring algorithm aversion but remains largely unexplored in the context of risk analysis and threat intelligence.

### 3.4 Domain Knowledge Versus Technical Expertise

Expertise enables analysts to make decisions in real time in a different way than novices (Kahneman and Klein 2009). Logg et al. (2019) found that novice forecasters tend to display the phenomenon of algorithm appreciation (as opposed to algorithm aversion).

A difference within the algorithm aversion literature is made between different kinds of expertise (Burton et al. 2020, Mahmud et al. 2022). The distinction is made between the concept of experience with the decision domain and algorithmic literacy. The former is about experience with the task at hand and according to some increases algorithm aversion even though others found no impact of task experience (Burton et al. 2020, Kawaguchi 2021). The latter concept is about experience in the interaction with algorithmic tools and could decrease algorithm aversion.

Experience with the decision domain has been proposed as the reason for an increase in algorithm aversion, as seen in Logg et al. (2019). Here, experts rely on their professional or specialized knowledge (Logg et al. 2019) and/or reliance on their schema (Okoli et al. 2016) instead of following the algorithm. This finding has been confirmed also by the series of experiments and interviews by Labunets et al. (2023) which showed that experts uses security knowledge to check that “nothing is forgotten” as opposed to novices who actually rely on it to find the actual solution.

Algorithmic literacy was proposed to diminish algorithm aversion because the person who has more experience interacting with algorithmic tools will have a better understanding on how to utilize and interpret algorithmic judgement (Burton et al. 2020). These findings are under the caveat that there is enough information about the quality of the algorithm output (Von Walter et al. 2022). In context of threat intelligence, however, the quality of information is uncertain, so we are interested to test whether experience has an impact on the perception of the intelligence source. For a more systematic account of the existing literature on explainability approaches for threat intelligence, we refer the interested reader to the review by Z. Zhang et al. (2022).

**Key Gap**: Previous research found that experience with the decision domain and algorithmic literacy have an effect on algorithm aversion. However, no previous study has looked at both forms of expertise/experience in a domain where quality of information is uncertain.

## 4 Research Questions

Based on the discussion above, our first research question is whether AI algorithms are considered to be better advisers for threat intelligence recommendations, even in presence of recommendations that are inconsistent with the cues present in the text.

**RQ1**. Is there an association between the underlying source of the recommendation (algorithmic versus human) or consistency of the recommendation (consistent versus inconsistent) and agreeing with a recommendation?

The first hypothesis is in line with the theory of perfect automation schema by Dzindolet et al. (2002) which claims that people predict “near perfect performance” from algorithms. In uncertain environments, near perfect performance was attributed to the human source (Dietvorst and Bharti 2020). Given the risk‐decisions are made in a group context, such as during safety analysis (Zhang and Liu 2022, Collier et al. 2023), expert judgment plays a pivotal role in risk decision‐making dynamics (Otway and von Winterfeldt 1992, Winter et al. 2022, Sniezek 1992) Indeed, a recent study shows that experts had different preferences when interpreting AI decision algorithm results (Paté‐Cornell 2024).

Given our population of MSc students, we postulated that the lack of expertise will leads to displaying the perfect automation schema (Rieger et al. 2023).

Hypothesis $H_{1,1}$
*The participants will disagree more with recommendations attributed to an AI algorithm compared to recommendations attributed to a human expert*. □

The second hypothesis aligns with the findings on cue inconsistency (Slovic 1966, Mandel et al. 2023), where more information cues were used when pieces of information (in our case, the threat analysis text and the advice by either the human or the machine) were consistent with one another. This effect was found in classical intelligence scenarios both for direct information (Mandel 2020) and for meta‐information (Mandel et al. 2023). The latter is precisely our case as the advice is a summary information.

Hypothesis $H_{1,2}$
*Participants will disagree more with the proposed recommendation when such recommendation is inconsistent with the cues in the text than when the recommendation is consistent with cues*. □

**RQ2**. What is the association between recommendations in terms of agreement, consistency, or underlying source and the overall perception of bias of the information?

The first hypothesis aligns with Birnbaum's finding in 1979 that agreeing with a source leads to perceiving that source as less biased (Birnbaum and Stegner 1979). This was especially the case when a source corroborated an already held judgment (Scharrer et al. 2019).

Hypothesis $H_{2,1}$
*Participants will attribute more bias when they disagree with the recommendation*.

The second hypothesis is symmetrical to the second hypothesis of RQ1 along with the utilization of information (Mandel et al. 2023).

Hypothesis $H_{2,2}$
*Participants will attribute more bias when they are given an inconsistent recommendation*.

In particular, Wallace et al. (2020) found that the perception of bias of a certain source decreased persuasion and source credibility *beyond* effects of untrustworthiness and lack of expertise. How this information combines with the literature on algorithm aversion and which direction a possible effect would take is not known to the best of our knowledge, so we only speculate that the participants will attribute more bias to an algorithmic source, testing the same direction as in $H_{1,1}$.

Hypothesis $H_{2,3}$
*Participants will attribute more bias to an algorithmic source than to a human source*.

The final RQ tries to address the key aspects of threat intelligence, namely: the highly uncertain environment where lack of familiarity with either the algorithm or the domain will attribute different characteristics to each sources (Rieger et al. 2023).

**RQ3**. What is the relationship between knowledge of the domain or knowledge of AI and the overall perception of the underlying source of the recommendation?

A recent academic study of commercial cyber threat intelligence vendors showed that they do not provide comprehensive and consistent reports (Bouwman et al. 2020). This is also part of the folklore as reported by the head of threat intelligence at Microsoft: “I read several threat intelligence reports daily. It is painfully obvious how the lack of analytic skill is harming the discipline” (Caltagirone 2014).

Hence, in line with the theory of perfect automation schema by Dzindolet et al. (2002), people with expertise in ML and their faults should not predict perfect performance from algorithms, whereas domain expert should not attribute perfect performance to the human source which they know to be occasionally mistaken (Dietvorst and Bharti 2020).

Hypothesis $H_{3,1}$
*The algorithmic source would be perceived as more biased by someone has more knowledge of AI algorithms than security knowledge than by someone who has more knowledge about security than AI algorithms*. □

## 5 Analysis Procedure

**RQ1**. To answer the first research question, we perform a logistic regression, where the binary variable y1 is represented by the disagreement of the participant with the recommendation: the value y1=0 means the participant agrees with the recommendation, instead y2=1 means the participants disagrees with the recommendation. In this way, we model the condition of silent assent with the suggested recommendation (Rieger et al. 2023).

We then have four different independent variables. The first variable x1 is the source of the recommendation, when x1=0, the source is human, x1=1 represents the AI recommendation. The variable x2 is the inconsistency of the recommendation and when x2=0, the recommendation is consistent, instead when x2=1, the recommendation is inconsistent. Once again we try to model as the 0 condition the absence of perturbing factors in the decision.

We use two additional control variables, x3 and x4 which describe the participants' experience. When x3=0, the participant does not have any experience in ML, instead x3=1 describes the case which the participant has some experience in ML. When x4=0, the participant does not have any security experience, instead we used x3=1 to represents a participant with some security experience. They are obtained by discretizing a number of scale items.

**RQ2**. To answer this research question, we perform a linear regression, where the scale variable y2 is represented by the overall perception of bias of the information. This value is obtained by taking the average of a number of items ranging from 1 to 5 and then recentered on 0 (subtracting 3 from the scale).

We then have five different independent variables. The variables {x1,x2,x3,x4} are the same as used in the logistic regression to answer RQ1. The new variable x5 would be the variable y1 of the logistic regression. Therefore, x5=0 means that the participant agrees with the recommendation, and x5=1 means that the participant disagrees with the recommendation.

**RQ3**. To test for this variable, we expect the coefficient of x3 (knowledge of AI) is positive and significant (larger bias) and the coefficient of x4 is negative (smaller bias).

*Equivalence as Alternative Validation for RQs*. Given the possibility of no effect, we apply the Two One‐Sided Test (TOST) procedure, originally proposed by Schuirmann (1981), which is widely used in pharmacology and food sciences to determine whether two treatments are equivalent within a specified range, defined either as an additive constant or a ratio (Food and Drug Administration 2001, Meyners 2012). For each of the RQ, in case we obtain a failure to prove the presence of an effect, we also test whether the two conditions are actually equivalent from the perspective of bias.

According to the guidelines set by the US Food and Drug Administration (FDA) and the European Medicines Agency (EMA), two drugs are considered equivalent if their respective distributions, x and y, satisfy the conditions x·ρ<y (one‐sided test) and $y<x\cdot \frac{1}{\rho }$ (the second one‐sided test), where ρ=0.8. To ensure a conservative approach, the final result is determined by taking the maximum of the two p‐values. The choice of the underlying directional test depends on the specific conditions, with either the Mann–Whitney *U* (MWU) test or the t‐test being used. In our study, we employed the MWU test. We set the significance level at p=0.05 and, to maintain a conservative evaluation, adopted the ρ value recommended by the FDA and EMA.

For example, to answer the third research question, we used TOST to test whether there is equivalence in the perceived bias among two sample groups: (1) participants with some AI experience but no security experience, (2) and participants with some security experience, but no experience in AI.

## 6 Ethical Approval

The experiment was part of two courses (in two consecutive years) taught by the experimenters at two different universities. Among other objectives, the courses had the intent to teach students about methods and measures of security experiments. The current experiment gave the students the opportunity to review and analyze the experiment and the obtained results. The students received grade points for the course for participating in the experiment.

The ethical procedure was followed and it determined that a full ethical review was not necessary. In particular, this was determined because (1) upfront, opt‐in consent was asked, (2) no personal or sensitive information was involved, (3) it did not pose potential risks to either participants or researchers, (4) the confidentiality of the participants was guaranteed by collecting data by GDPR compliant tool and removing their details before processing the data for the analysis, (5) the participants were thoroughly debriefed afterward (they actually had full access to the anonymized data to use for their own report). Finally, (6) the incentives to participate were minimal: there was no monetary compensation, and the participants received a compensation in terms of coursework's bonus. Such value was minimal (less than 2% of the final grade), and the participants could deny the consent to use data for research, and still obtain the participation bonus. Besides name and student number which were necessary to grand the coursework's bonus, we did not collect any other personal information. Those personal identifiable information have been deleted from the dataset before moving to data analysis.

## 7 Reports Chosen as Experimental Artifacts

### 7.1 Data Source

The Dutch National Cyber Security Centre (NCSC) is the governmental single point of contact when it comes to cyber threats and incidents and has the legal obligation to analyze and research cyber threats and incidents (Kamara et al. 2020). The NCSC is comprised of experts and works together with other expert organizations. NCSC advisories are governmental reports with the purpose of describing what a specific vulnerability entails and what could potentially happen if this vulnerability is exploited (NCSC 2023c). The advisories themselves provide a “direct action perspective” for organizations (NCSC 2023a). The advisories include a risk assessment of the likelihood/chance of exploitation (“Kans”) and the impact/damages that possible exploitation could entail (“Schade”) (NCSC 2023c). These assessments result in a categorical value of low, medium, or high. The likelihood judgments are established by using a classification matrix (NCSC 2023b). See Table 1 for an overview of the likelihood classification matrix.

**TABLE 1:** Likelihood classification matrix NCSC.

Question	Option 1		Option 2		Option 3	
Is the vulnerability present in the standard configuration/installation?	No	1	Unclear/Yes	3		
Is there Exploit Code available?	None	1	Proof‐of‐Concept (PoC)	4	Exploit	6
Are there any technical details available?	None	1	Somewhat	2	Completely	3
Required Access?	Physical	1	LAN/Direct Environment	4	Internet	6
Required credentials?	Admin	1	User	2	None	4
How complex is it technically to exploit the vulnerability?	Complex	1	Average	2	Simple	3
Is there user interaction necessary?	Complex	1	Simple	3	None	4
Is the vulnerability exploited in the wild?	No	1	Limited scale	2	Large scale	3
Is the expectation that the vulnerability will be exploited in the short term?	No	1	Yes	3		
Is there a solution available?	Older than 2 months	1	Up to 2 months old	2	None	3

*Note*: **NCSC likelihood matrix** (NCSC 2023b). The higher the score in matrix, the higher the risk. Buckets for the categories are 10–18 for a low assessment, 19–27 for a medium assessment, and 28–38 for a high assessment.

### 7.2 Selection Criteria of Original Reports

The chosen reports and the described vulnerabilities are presented in Table 2. Full reports were selected based on whether there was a transition from medium risk of chance of exploitation to a high risk. This transition was based on new updates that were viewed as pieces of threat intelligence. Only reports were chosen that either in their description or in the attached pieces of threat intelligence mention one specific vulnerability (some reports mention multiple vulnerabilities). Furthermore, reports were only chosen if they had a high impact/damages risk classification. This choice was made to operationalize the step from medium/high priority to the highest and most critical priority. The first eight reports that met these conditions are reported in Table 2.

**TABLE 2:** Chosen reports and main CVE.

ID	Report	Main CVE
1	NCSC‐2023‐0428	CVE‐2023‐38035
2	NCSC‐2023‐0277	CVE‐2023‐20887
3	NCSC‐2023‐0282	CVE‐2023‐27997
4	NCSC‐2022‐0368	CVE‐2022‐22972
5	NCSC‐2022‐0334	CVE‐2022‐1388
6	NCSC‐2022‐0056	CVE‐2022‐23131
7	NCSC‐2023‐0256	CVE‐2023‐2868
8	NCSC‐2023‐0346	CVE‐2023‐29300

#### 7.2.1 Processing of Original Reports

To maximize ecological validity while keeping construct validity and providing a report that is easy to process in the allotted time, we have limited the simplification of the text to a minimum.

The full reports were abbreviated by removing details on version numbers (which could span for several sentences), and other irrelevant information. The specific sentences summarily revealed the NCSC classification were also removed as they would provide participants the “ground truth” (e.g., “The advisory has been changed to HIGH/HIGH”). Similarly, information on CVSS scores was removed from the text as it would provide this additional ground truth information.

All descriptions and pieces of threat intelligence that were attached to the risk classification were kept. The source of the threat intelligence, when available, were always presented (e.g., “The FBI reports that…”).

Since reports are updated in time, the additional sentences added after the initial risk classification of the first published report were removed from the main text and presented as background threat intelligence with a date. The rationale is that the sentences reflected pieces of obtained threat intelligence that were actually unknown at the time of initial risk classification and should be explicitly presented as such. If the experiment were executed across days with new information being posted into the system, the participants would have directly perceived this additional threat intelligence as “what's new.” We simply presented them as change log in Background Threat Intelligence.

**FIGURE 1:**
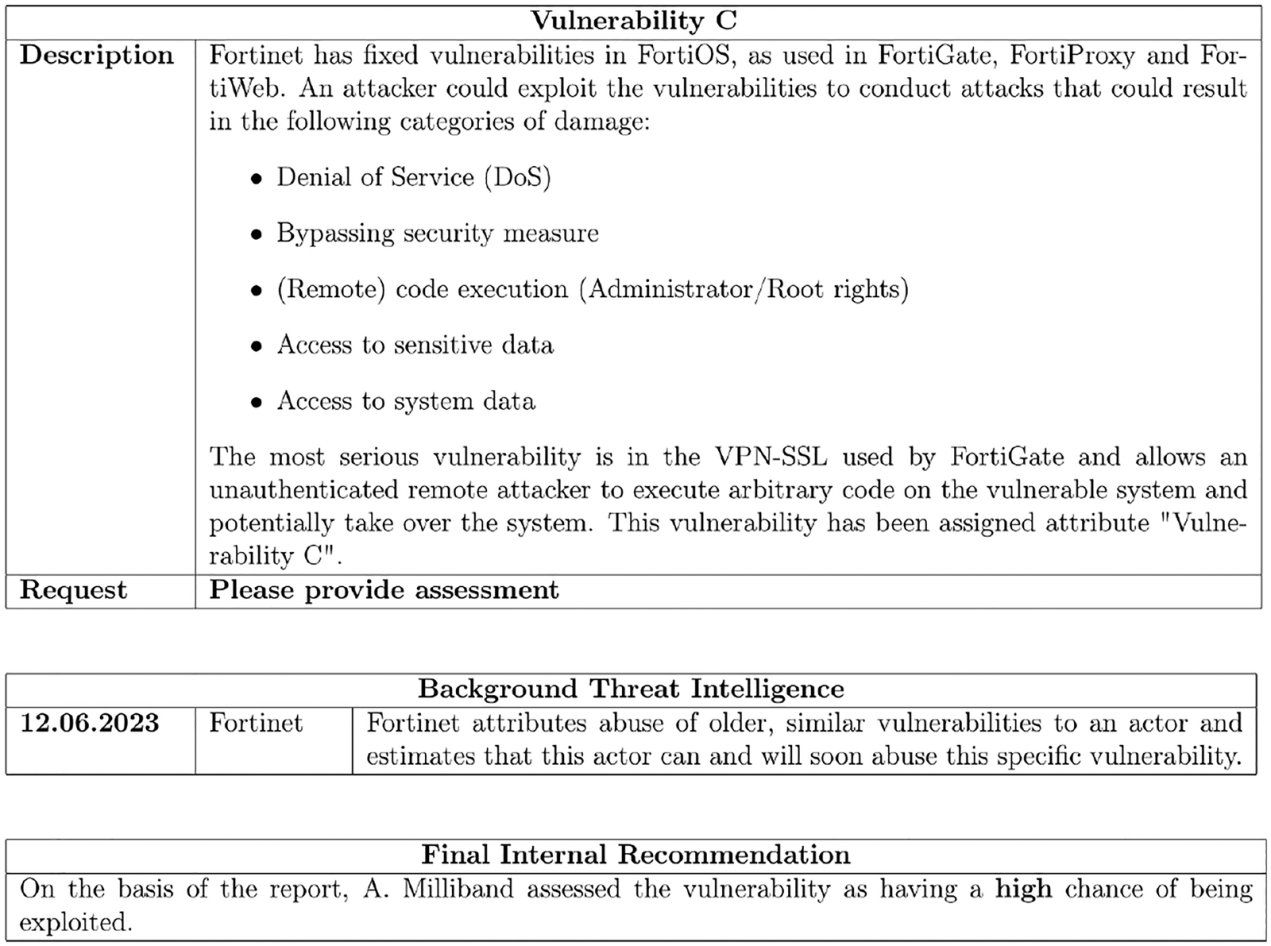
Example of report with original recommendation according to the NCSC is high risk, and the source is human.

The final result is presented in Figure 1.

*A limitation of the real data is that reports are never downgraded*. So once something is high, it stays high. Hence, for report with medium risk, we could only use reports that show all pieces of threat intelligence that were available for medium risk to accompany medium reports. For high reports, we could use both threat intelligence in which the report was high from the very beginning and pieces of threat intelligence were taken that updated the risk classification from medium to high. We do not have pieces of threat intelligence evidence in which an initial high risk according to the NCSC is downgraded to medium.

### 7.3 Experimental Manipulations

In addition to the description and threat intelligence, a recommendation is added to the reports. The recommendations are the operationalization of the first two different conditions of the experiment: source and consistency. This is further described in the experimental design in Section 8. Figure 1 shows the third abbreviated high report with a human recommendation that is inconsistent with the NCSC recommendation. The information is hidden to the participant, they do not know what the original recommendation of NSCS was, as it was removed from the report. The presentation of the source as an AI or human sources does not essentially changes the ecological validity of the recommendation. To obtain some reports in which the recommendation is “wrong,” we have changed the recommendation (switching from high to medium or from medium to high). This is necessary to provide variety of scenarios and to make sure that participants are confronted with cases in which choice is wrong and therefore they might to disagree with it. Reporting only “right” recommendation would violate the construct validity of the experiment. See Appendix for the other conditions with respect to Report 3.

## 8 Experiment Design

### 8.1 Target Populations

The intended novice population was computer science master students. The participants, recruited from two different universities provide a mix of two different expertise. The first group consisted of students that had previous experience with risk assessment in their curriculum. The second group consisted of students that had previous experience with ML. In both cases, the students had English proficiency (both masters were taught in English) and a background in computer science as a BSc in CS was a prerequisite for enrollment.

### 8.2 Procedure

The participants received a *specific training* activity of before the experiment, which included an introduction to threat intelligence (1.5 h) and a lecture on vulnerability risk assessment (1.5 h). In addition, the participants were trained on using the classification matrix for likelihood and impact/damages of the NCSC (translated in English) and received a short walkthrough video was given with one example task (not part of the actual experiment) and two short exercises. The goal of these training activities was to ensure that the students had the ability to perform the task. Previous work in experimentation with students showed that under the correct conditions, students were able to perform tasks at a level comparable to experts (Allodi et al. 2020, Salman et al. 2015).

The experiment took place 1 *day after the training* activity and consisted of a maximum of 2 h. During the experiment, participants had access to all the training material and they could take breaks, finish, or stop at any time. The experiment was held in a room where at least one supervisor was present. The role of the supervisor was to help with technical difficulties but not answer any questions relating to the content of the assessments.

At the beginning of the experiment, the students were asked to give *informed consent*. Thereafter, the participants were able to read the task description and answer questions about previous experience. Then, the students were randomly assigned to 1 of 12 groups. These groups reflected the different order of manipulations to ensure a balanced design (i.e., the measurements obtained in each condition are roughly equal). An overview of the *design of the groups* can be observed at the top of Table 3.

**TABLE 3:** Taguchi balanced design: Groups and order manipulation.

First measurement/SecA								
Report	1	2	3	4	5	6	7	8
**ID group**								
A	H‐C‐M	H‐I‐H	A‐C‐H	A‐I‐M	A‐I‐M	A‐C‐H	H‐I‐H	H‐C‐M
B	H‐I‐H	A‐C‐H	A‐I‐M	H‐C‐M	H‐C‐M	A‐I‐M	A‐C‐H	H‐I‐H
C	A‐C‐H	A‐I‐M	H‐C‐M	H‐I‐H	H‐I‐H	H‐C‐M	A‐I‐M	A‐C‐H
D	A‐I‐M	H‐C‐M	H‐I‐H	A‐C‐H	A‐C‐H	H‐I‐H	H‐C‐M	A‐I‐M
E	H‐C‐M	A‐C‐H	A‐I‐M	H‐I‐H	H‐I‐H	A‐I‐M	A‐C‐H	H‐C‐M
F	H‐I‐H	A‐I‐M	H‐C‐M	A‐C‐H	A‐C‐H	H‐C‐M	A‐I‐M	H‐I‐H
G	A‐C‐H	H‐C‐M	H‐I‐H	A‐I‐M	A‐I‐M	H‐I‐H	H‐C‐M	A‐C‐H
H	A‐I‐M	H‐I‐H	A‐C‐H	H‐C‐M	H‐C‐M	A‐C‐H	H‐I‐H	A‐I‐M
I	H‐C‐M	A‐I‐M	A‐C‐H	H‐I‐H	H‐I‐H	A‐C‐H	A‐I‐M	H‐C‐M
J	H‐I‐H	H‐C‐M	A‐I‐M	A‐C‐H	A‐C‐H	A‐I‐M	H‐C‐M	H‐I‐H
K	A‐C‐H	H‐I‐H	H‐C‐M	A‐I‐M	A‐I‐M	H‐C‐M	H‐I‐H	A‐C‐H
L	A‐I‐M	A‐C‐H	H‐I‐H	H‐C‐M	H‐C‐M	H‐I‐H	A‐C‐H	A‐I‐M

Second measurement MLA								
Report	1	2	3	4	5	6	7	8
ID group								
A	H‐C‐H	H‐I‐M	A‐C‐M	A‐I‐H	A‐I‐H	A‐C‐M	H‐I‐M	H‐C‐H
B	H‐I‐M	A‐C‐M	A‐I‐H	H‐C‐H	H‐C‐H	A‐I‐H	A‐C‐M	H‐I‐M
C	A‐C‐M	A‐I‐H	H‐C‐H	H‐I‐M	H‐I‐M	H‐C‐H	A‐I‐H	A‐C‐M
D	A‐I‐H	H‐C‐H	H‐I‐M	A‐C‐M	A‐C‐M	H‐I‐M	H‐C‐H	A‐I‐H
E	H‐C‐H	A‐C‐M	A‐I‐H	H‐I‐M	H‐I‐M	A‐I‐H	A‐C‐M	H‐C‐H
F	H‐I‐M	A‐I‐H	H‐C‐H	A‐C‐M	A‐C‐M	H‐C‐H	A‐I‐H	H‐I‐M
G	A‐C‐M	H‐C‐H	H‐I‐M	A‐I‐H	A‐I‐H	H‐I‐M	H‐C‐H	A‐C‐M
H	A‐I‐H	H‐I‐M	A‐C‐M	H‐C‐H	H‐C‐H	A‐C‐M	H‐I‐M	A‐I‐H
I	H‐C‐H	A‐I‐H	A‐C‐M	H‐I‐M	H‐I‐M	A‐C‐M	A‐I‐H	H‐C‐H
J	H‐I‐M	H‐C‐H	A‐I‐H	A‐C‐M	A‐C‐M	A‐I‐H	H‐C‐H	H‐I‐M
K	A‐C‐M	H‐I‐M	H‐C‐H	A‐I‐H	A‐I‐H	H‐C‐H	H‐I‐M	A‐C‐M
L	A‐I‐H	A‐C‐M	H‐I‐M	H‐C‐H	H‐C‐H	H‐I‐M	A‐C‐M	A‐I‐H

*Note*: H‐C‐M Recommendation given by human, consistent with the report, and the original NCSC likelihood assessment of the report was medium H‐C‐H Recommendation given by human, consistent with the report, and the original NCSC likelihood assessment of the report was high. H‐I‐M Recommendation given by human, inconsistent with the report, and the original NCSC likelihood assessment of the report was medium. H‐I‐H Recommendation given by human, inconsistent with the report, and the original NCSC likelihood assessment of the report was high. A‐C‐M Recommendation given by algorithm, consistent with the report, and the original NCSC likelihood assessment of the report was medium. A‐C‐H Recommendation given by algorithm, consistent with the report, and the original NCSC likelihood assessment of the report was high. A‐I‐M Recommendation given by algorithm, inconsistent with the report, and the original NCSC likelihood assessment of the report was medium. A‐I‐H Recommendation given by algorithm, inconsistent with the report, and the original NCSC likelihood assessment of the report was high.

The randomization was followed by the likelihood assessment of the eight abbreviated reports. The participants were asked to explain their answers. These questions were followed by manipulation and attention checks.

See Appendix for an overview of the attention checks per abbreviated report. Thereafter, questions about the report in question and the current assessment were answered. When all reports were assessed, questions were displayed about the quality and usability of the different sources.

### 8.3 Experimental Manipulation Variables and Dependent Variables

See Table A.1 for an overview of the variables in the experiment. For RQ1, the variable *Source* reflects the main condition where a recommendation is either “given” by a human or an AI source:

- A. Milliband, a senior risk assessment human analyst with over 15 years of experience in the field
- HackHunter, an established ML algorithm with high accuracy scores

**TABLE 4:** RQ1—Logistic regression results.

*y* (1 = Disagreement)					
	Coeff.	Std. Err	*p*‐value	[0.025	[0.095]
x1 Source	0.6519	0.195	0.001	0.269	1.035
x2 Inconsistency	0.3523	0.195	0.071	−0.030	0.735
x3 ML Exp.	0.1274	0.213	0.549	−0.289	0.544
x4 Security Exp.	−0.7078	0.237	0.003	−1.173	−0.243
**Pseudo R‐squared**			**Log‐Likelihood**		
0.03778			−297.63		

**TABLE 5:** RQ2—Regression results.

*y*2 = Bias					
	Coeff.	Std. Err	*p*‐value	[0.025	[0.095]
x1 Source	0.0051	0.068	0.941	−0.129	0.140
x2 Inconsistency	0.0346	0.068	0.611	−0.099	0.168
x3 ML Exp.	−0.0425	0.074	0.565	−0.187	0.102
x4 Security Exp.	−0.0007	0.080	0.993	−0.157	0.156
x5 Disagreement	0.5831	0.070	0.000	0.445	0.721
**Adjusted R‐squared**			**F‐statistic**		
0.133			14.86		

**TABLE 6:** Summary of findings and implications.

Main findings	Risk researcher	Practicing risk analyst
When the source of the recommendation is algorithmic, participants disagreed with the recommendation more.	A possible implication is that, in absence of quality information about the algorithm, a novice technical understanding is enough to instill algorithm aversion. This should be taken into account when tailoring algorithmic advice to different populations.	Following the recommendation of Paté‐Cornell (2024), the factor of the source type should become as transparent as possible, especially for novice analysts working in threat intelligence.
Recommendations that are consistent with the information cues lead to equal perception of bias compared to the recommendations that are inconsistent.	The implication is that enough information should be presented in reports for a novice population to be able to retrace the steps. Researchers could test the border where additional information is just right (instead of too little or too much).	Analysts using AI to support their risk decisions should adopt such technologies with care, as they tend to provide inconsistent advice (Collier et al. 2023).
Participants with more security expertise agreed more with the recommendation.	Replications including senior experts could be an interesting avenue for future research to see whether the latter might be more sensitive to “wrong” recommendations presenting interaction effects.	Making judgments based on limited information might rely more on pattern recognition than on analysis (Gigerenzer and Gaissmaier 2011, Winter et al. 2022, Labunets et al. 2023)
The higher the disagreement the more the participants perceived bias of the source and recommendation	This finding confirms the findings of Logg et al. (2019) in that a possible factor in the perceived bias is corroboration with own decision.	One recommendation would be to make the levels of individual analyst disagreement explicit and transparent before the final risk decision takes place.
Equivalence in perceived bias	Replications and new research in algorithm aversion under uncertain risk decision‐making with AI should be considered.	Training practitioners to focus on quality of information but also reflecting on the source, should be considered, as well as implementing checks and balances also on algorithmic decisions.

Additionally, the variable *Consistency* represents the main condition where the likelihood recommendation is manipulated while keeping the text identical to the ground truth. The recommendation is represented with the categories “medium” and “high,” respectively.

Additional variables are taken as independent variables in the analysis. For RQ2, the discussed experimental manipulated variables are present but the previously dependent variable of *Agreement with recommendation* is added to the independent variables. For RQ3, the difference in groups is taken as the independent variable.

Dependent variables are collected in line with the three research questions. For RQ1, the binary variable *Agreement with recommendation* is measured as a behavioral proxy to trust (Prahl and Van Swol 2017). For RQ2, the *Perception of bias of information* is measured as the self‐reported perception of bias of the report and recommendation. This variable is operationalized as an aggregated Likert scale consisting of four items. For RQ3, the dependent variable is the *Difference score* between the overall perception of the human source and the overall perception of the algorithmic source. If the score is above 0, the difference is in favor of the human source. If the score is below 0, the difference is in favor of the algorithmic source. The difference score is calculated based on the perceived overall usability and quality for each source.

### 8.4 Control Variables

A number of other variables are collected to control the internal and construct validity of the results of the experiment. Assessment validation variables are collected to verify whether the vulnerability assessment will be completed on the basis of the information in the report. The purpose of the *Manipulation check* question is to measure whether the participant is aware of the recommendation source. Two questions about the reported information have the purpose to check whether the participant read the information (Variables for *Attention checks*, see Appendix).

Experiment validation variables are collected to verify whether the experimental process has been performed correctly. The purpose of the *task understanding* item is to verify whether the participant had a clear understanding of the task at hand. The participant is asked about the *task time* to see whether the participant had enough time to finish the task. In addition, the purpose of the *task training* item is to validate that the provided training materials were sufficient for completing the task.

Background variables are collected to test whether the planned differences among groups is actually realized in the sample. Two background variables are measured for security. First, *Knowledge of vulnerability risk assessment* is measured to gauge the participant's amount of previous experience with vulnerability risk assessment. Second, *Knowledge of reading threat intelligence* is measured to gain insight into the participant's amount of previous experience with reading threat intelligence reports. To capture experience with algorithms, *Knowledge of using ML algorithms* is measured to gauge the participant's amount of previous experience with using ML algorithms. In addition, *Knowledge of developing ML algorithms* is measured to gain insight into the participant's amount of previous experience with developing ML algorithms.

## 9 Study Execution

### 9.1 Participants

Our population is composed by a total of 57 computer science MSc students. We can identify two main groups: (1) one composed of participants with no experience in the security field (60% of the population) (2) and the other one with no experience in AI (47% of the population). A total of 29 participants (51%) said that they have some security experience, and 53% of the population has some experience in AI.

### 9.2 Experimental Controls

One participant failed one manipulation check from a total of eight manipulation checks. Five participants failed 1 attention check from a total of 16 attention checks. The authors analyzed the explanations given by the participants for the assessment reflected by these data points. These explanations were deemed plausible by the researchers and no participant was removed.

**FIGURE 2:**
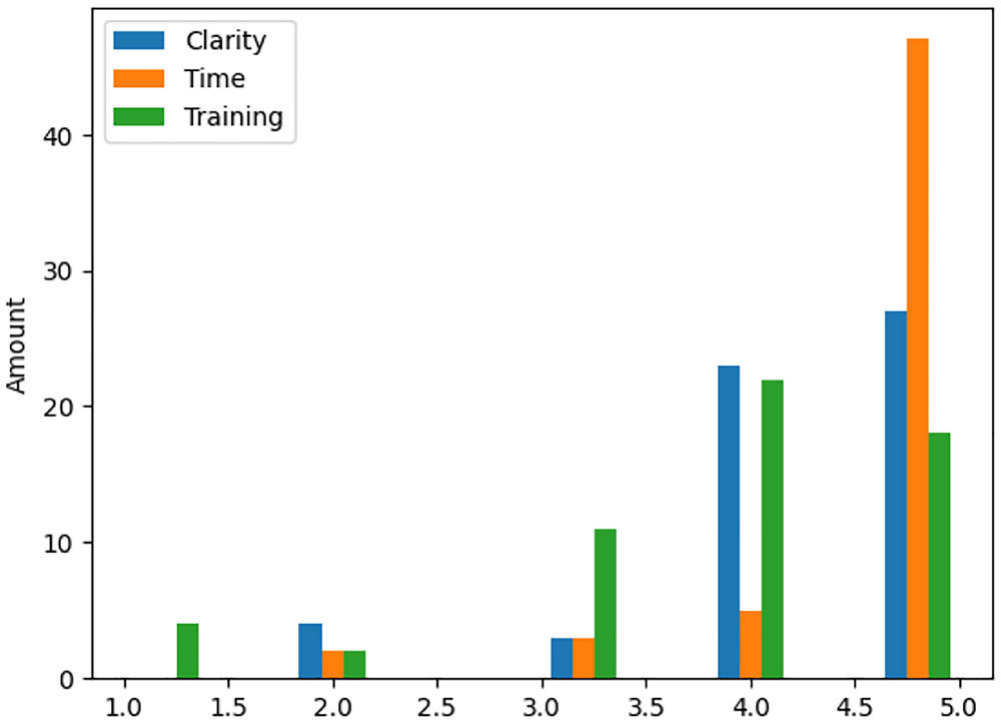
Graphical representation of the scores of the three experiment validation variables (clarity, time, and training) in relation to the amount of participants.

To test the validity of the experiment, in terms of the ability of participants to understand and complete it, the self‐reported responses of control questions were tested. These experimental validation variables are presented in Figure 2.

A validation variable was considered sufficient if it scored 4 or higher. Task time was considered sufficient by 95% of the participants. Task understanding was considered sufficient by 89% of participants. Task training was considered sufficient by 70%, while only 10% of the participants thought that the training was insufficient (i.e., gave a score less than 3). Therefore, it is assumed that all three controls are sufficiently met.

The analysis confirms that the construction of the experimental groups is appropriate for the purpose of the study.

## 10 Results

In this section, we report the results of our investigation. In Section 10.1, we describe the findings we obtained to answer RQ1, instead in Sections 10.2 and 10.3, we answer respectively to RQ2 and RQ3. The statistical analysis was performed according to the choice of the analysis procedure described in Section 5.

### 10.1 RQ1

Table 4 reports the results we obtained from the linear regression that we performed using the variable y1 to describe the disagreement (when y1=1, the participant disagree with the recommendation). Each row of the table is a different dependent variable of the regression.

To verify if H1.1 is satisfied, x1 (which is 1 when the source is AI) has to be positive and statistically significant, which is what we observed from the results we obtained; therefore, the data support H1.1, and we can conclude that the participants tend to disagree with the recommendation when the source is AI.

Finally, we observe that the variable x2 is positive and not statistically significant; therefore, the data do not support H1.2; therefore, it seems the inconsistency with the original NCSC recommendation (hidden from the participant) do not have an impact.

We also observed that the more experience the participants have in the security field, the less they disagree with the recommendation. We also tested for interaction effects between people with security experience and the disagreement with the recommendation was actually “wrong” (inconsistency = 1) as it was not what the NCSC would have recommended it. However, we did not find any significant interaction effect.

### 10.2 RQ2

Table 5 reports the results we obtained from the regression that we performed using the variable y2 representing the overall perception bias of the recommendation. We adjusted this variable computing y2=y2−3 and the resulting value was then used in the regression. As for Table 4, each row is a different dependent variable of the regression.

To verify if H2.1 is satisfied, the variable x5 (that when it is equal 1 means that the participant disagrees with the recommendation) has to be positive and statistically significant. As we observe from the results reported in Table 5, the data support H2.1.

In contrast, the data do not support H2.2 and H2.3 as both x1 and x2 are not statistically significant. Therefore, we performed a TOST to verify if there is an equivalence between the two sample groups. For the case of H2.2, we considered the inconsistency variable x2 and we obtained $U_{lower\_bound}=13607,p=2.04\cdot 10^{-18},U_{upper\_bound}=16505,p=2.19\cdot 10^{-11}$; therefore, the group of participants that have an inconsistent recommendation is equivalent to the one with a consistent recommendation. For the case of H2.3, we considered the source variable x1 and we obtained $U_{lower\_bound}=13757,p=5.27\cdot 10^{-18},U_{upper\_bound}=16324,p=9.04\cdot 10^{-13}$; therefore, the group with human source is equivalent to the algorithmic source.

### 10.3 RQ3

To answer RQ3, we performed TOST to verify if there is an equivalence between the two sample groups: (1) participants with some experience in the security field, but no experience in AI and (2) participants with some experience in ML, but no experience in the security field. The result is $U_{lower\_bound}=1869,p=3.31\cdot 10^{-05},U_{upper\_bound}=1901,p=4.59\cdot 10^{-05}$; therefore, the two groups are statistically equivalent, and the data do not support H3.1. It actually supports *the opposite of the hypothesis*: some experience does not make a difference.

### 10.4 Robustness Checks

Possible noise factors have been analyzed to increase the robustness of the results in Appendix.

## 11 Threats to Validity

*Order Effects of Algorithmic Errors*. The overall perception of a source is dependent on the position of the observed errors of algorithmic performance. Observing an algorithm make an error at the beginning could lead to greater algorithm aversion (Dietvorst et al. 2015). The current study may be impacted by order effects of algorithmic errors. However, the present research follows a balanced design seen in Table 3 which controls for these effects, since there is an equal amount of randomly assigned groups encountering their first algorithmic judgment as inconsistent (group ID D, F, H, I, J, and L) as well as consistent (group ID A, B, C, E, G, and K). The general theory shows that if scenarios (threat descriptions in our case) are sufficiently different, the impact is minimal (Massacci et al. 2024).

*Self‐Report Scales*. The current study uses self‐report scales to measure two outcome variables: overall perception of bias and perception of source. The idea that sources and information can be biased is prevalent in the threat intelligence community (Belton and Dhami 2020). The items concerning bias were therefore constructed to be taken at face value (i.e., “The overall final recommendation looks biased”). No external validity tests have been performed. One possibility is that the items only are not an accurate measurement and thus, skew the results. Evaluating cyber threat intelligence sources can be done based on the complex concept of information quality of the source (Schaberreiter et al. 2019). In the current study, that information would be the recommendation. Currently, the concept is measured using one item to capture the concept at face value, and one item to capture the continued usability. More sophisticated items might be necessary to capture the entire concept.

*Risk of Differentiated Measures*. The experiments have been executed with two different groups at two different times and places. In addition, there were three nonstructural changes made between the two measurements. These changes were based on lessons learned from conducting the first experiment with the SecA group and consisted of: an additional extra item measuring impact assessment, the location of the manipulation checks, and an extra set of questions measuring expert level. Changes occurring with differentiated measures can introduce confounding factors. However, the first experiment was replicated with the intention to keep the differences to a minimum by using the same training and lecturers, the same experimental design (including number of rooms), and the same experimental objects. Controlled by the fact that not much was changed, the same scripts were able to run in the same way as the first experiment.

*Choice of Population*. We acknowledge that the background and prior experience of participants may affect the validity of experimental results. However, several studies with students have reported encouraging results (Chong et al. 2021, Naiakshina et al. 2017, 2018, Rong et al. 2012). In particular, Salman et al. (2015) compared the performance of students and professionals to determine to what extent students can serve as valid proxies for professionals in SE research. Their findings indicate that, when confronted with a task for the first time, the performance of both groups tends to be comparable. Furthermore, Tahaei and Vaniea (2022) conducted an experiment investigating programming skills, privacy and security attitudes, and self‐efficacy in secure development among participants recruited from a student mailing list for CS and four crowd‐sourcing platforms (Appen, Clickworker, MTurk, and Prolific). Their results show that 89% of the CS students correctly answered all programming skill questions, compared to only 27% of the participants on crowd‐sourcing platforms. However, we acknowledge that the findings shown in this study are considered preliminary results due to the choice of population; therefore, more experiments should be conducted, including with cybersecurity experts, to further investigate the results obtained and fill the gap with real‐world scenarios.

*Measuring Experience in Students*. Measuring domain experience in a student population can cause difficulties since the spread of expertise might not exist (i.e., the group is too homogeneous and represents only the novice perspective). Additionally, experience might only translate to knowledge of the decision domain without the actual practice (Labunets et al. 2023). This is also the case in the present experiment where most participants have either no experience or university experience with the decision domain. Although this experiment does include an actual item measuring the self‐reported previous experience with vulnerability risk assessment, an additional proxy measure was introduced to mitigate this issue. The proxy used is the experience in reading threat intelligence reports. The proxy shifts the focus of experience with “real” decisions in the domain to experience with just one facet, the reading of the reports. To generalize our results to a broader range of security professionals, it can only be drawn with more experiments.

*Sample Size*. Given our small sample size, it is possible that the failure to produce significant results might have been due to lack of power. To mitigate this risk, we have also tested for statistical equivalence among conditions doing a number of two one‐sided tests. In some cases, this allowed to prove that there was really no difference between the conditions. In other cases also, the TOST was not significant and more experiments would be needed.

*Selection of Original Reports*. At first, the entire advice was fully derived from the NCSC which preserved all key elements of ecological soundness. The main experimental manipulation (the AI vs. human source) was textually minor as it just changed the name of the source and therefore could not impact the soundness of the advice. The consistency manipulation may indeed affect the ecological validity but cannot be eliminated while keeping the construct validity of the experiment: To assess potential bias and disagreement, we need some scenarios in which the recommendation is *wrong*. If the recommendation was always right, participants would eventually tend to agree with it irrespective of the source. Assuming that the NCSC knows what they are doing when giving advice on the Internet, their final recommendation is likely *right* (at least to the best of expert knowledge). Therefore, the only way to have a wrong recommendation is to actually change it.

*Experimental Manipulation*. The current manipulation oversimplifies how professionals build trust in recommendations by focusing on the label of the source. For example, factors such as accumulated performance (Dietvorst et al. 2015) and peer discussion within specific team dynamics (Chun and Park 1998) are not taken into account. Therefore, it is not clear whether participants responded to the label itself or assumptions about quality, transparency, or accountability. Although this choice was made for experimental clarity, future research is necessary to explore these nuances.

## 12 Implications for Research and Practice

An overview of the main findings and the implications for research and our recommendations for practice are found in Table 6.

Policywise, our research pinpoint at two major gaps in the US industrial policy recommendations for cyber threat intelligence sharing (NIST‐800‐150; Johnson et al. 2012):

- while it is recommended to investigate the technical skills and proficiencies of the target communities (“What are the technical skills and proficiencies of the community members?” (Johnson et al. 2012)), there is no concrete guidance on how to present CTI to different populations.
- the existing guidelines encourage the organizations to find multiple sources of intelligence to confirm its credibility (like, internal sources and open sources). But, to date, there is no guidance regarding the triangulation process when sources used in practice are of fundamentally different types (expert vs. AI‐based).
- assuming that participants would be skeptical of AI suggestions is not necessarily supported, so *check and balances by independent algorithms* should be foresee also for algorithmic decisions

## 13 Conclusion and Future Work

More automated analysis in vulnerability assessment and cyber threat intelligence is the new trend pushed by industry and governments alike. To test how the difference (human vs. AI) of cyber threat intelligence recommendations might be perceived by users, we designed a controlled experiment. We have asked n=57 MSc students in computer science with a varied experience in ML and security to assess a number of cyber threat intelligence recommendations extracted from original reports from the Dutch National Cyber Security Center, for a total of 456 data points.

Our findings revealed that participants tended to disagree with the recommendation when it was coming from AI showing a general tendency of algorithm aversion. While expertise on ML did not have any impact, we found that participants with more security expertise tended to agree with the recommendation. In contrast to simplistic narratives of algorithm aversion, we found that the perceived bias was statistically equivalent whether the recommendation was coming from a human or from an AI (TOST). In a nutshell, *our participants are algorithm neutral* when it comes to judging bias, they exhibit neither aversion nor appreciation in terms of bias. This is in line with the ideas of Wallace et al. (2020). We only measured perceived bias when participants disagreed with the recommendation (irrespective whether it was human or AI). This is to be expected and it is re‐assuring in terms of the validity of the experiment that what is obvious is also statistically confirmed.

These results provide insight on the possible impact of introduction on AI on rank‐and‐file Tier 1 SOC analysts. Although the current findings provide a useful addition to ongoing discussions about algorithm aversion, it is important that the results are interpreted with modesty. Students and professionals do not face the same decision‐making pressures, contextual familiarity, or accountability constraints. Research shows that workload, complexity of the decision, complexity of the decision domain, and cognitive strain influence the extent to which decision makers rely on automated advice (Chugunova and Sele 2022, Kaufmann et al. 2023). In addition, organizational influences like team dynamics, the placement of automation within the organizational structure, and the degree to which a decision maker is held accountable for the task at hand influence decision‐making and reliance on automated advice (Chun and Park 1998, Selten and Klievink 2024, Mahmud et al. 2022). Some research showed that the perception of more accountability leads to a reduction in automation bias (Goddard et al. 2012) while others challenged this finding (Skitka et al. 2000). On top of this, factors such as different political and bureaucratic systems influence how similar automated tools can yield different results within an organizational context (Giest and Samuels 2023). Another big difference when performing the current study with experts lies in measuring the conceptual difference between social trust and confidence. Social trust is based on perceived similarities in values and intentions, while confidence is based on experience and/or evidence (Siegrist 2021). As discussed in Siegrist (2021), it is possible that a similar question could measure social trust in novices while measuring confidence in experts. To validate and generalize our finding, replication experiments must be performed with a larger and more diverse population (e.g., including professional security experts and data scientists) and consider to expand the current method with follow‐up questions to uncover the differentiation between trust and confidence. However, some of the aforementioned factors are difficult and sometimes impossible to recreate within a laboratory setting but are vital in understanding the phenomenon. Future research could therefore focus on follow‐up studies in a field setting. Furthermore, as discussed in Section 11, the experimental manipulation was kept simple for experimental clarity. Future work might explore more realistic instantiations of AI systems (e.g., varying explainability) to explore the different nuances. An interesting theoretical study would be to analyze what happens when likelihood of exploitation is downgraded. Such study cannot be derived from the real data as no such a thing as a risk downgrade can be found in government‐managed data sources of cybersecurity vulnerabilities (for the obvious reasons). Finally, new balanced designs should be tested to capture whether the order in which inconsistent judgments are received influences the overall perception of the source.

## Author Contributions

**Sarah van Gerwen**: conceptualization, methodology, software, investigation, writing – original draft, writing – review and editing. **Aurora Papotti**: methodology, software, validation, investigation, data curation, writing – review and editing, visualization. **Katja Tuma**: conceptualization, validation, writing – review and editing, supervision, project administration, funding acquisition. **Fabio Massacci**: conceptualization, methodology, validation, writing – review and editing, supervision, project administration, funding acquisition.

## Funding

This study was supported by HEWSTI.

## Figures and Tables

**FIGURE B.1:**
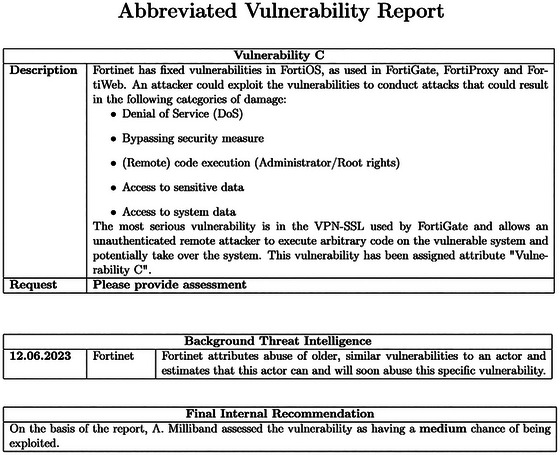
Report 3: Examples of the artifact in the different conditions for human.

**FIGURE B.2:**
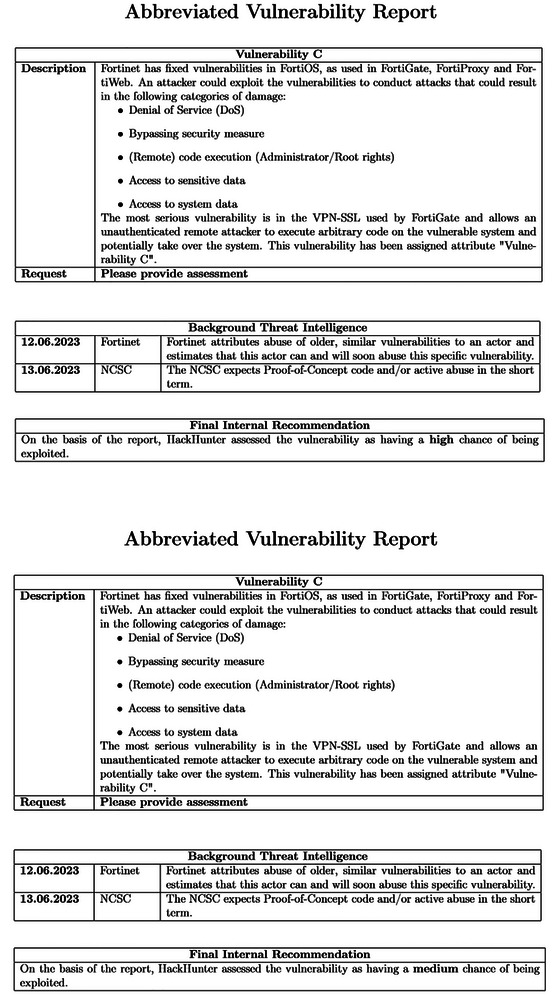
Report 3: Examples of the artifact in the different conditions for AI.

## Additional Information on the Experiment

For an overview of the variables in the experiment, see Table A.1. Table A.2 shows the two attention checks included for each report. To increase the robustness of the results, the association between level of report and agreement with the recommendation was analyzed. This association could serve as a possible confounder for the significant association of source and agreement in the MLA group. This is due to the operationalization of consistency in combination with the chosen report levels (medium vs. high). The operationalization resulted in a situation where the recommendation attributed to a certain source had the same value across the source condition within a group (e.g., a consistent report for a medium‐level report and an inconsistent report for a high‐level report both resulted in a medium recommendation). A chi‐squared test showed no significant association between level and agreement in the SecA group (*X2* (1, *N* = 218) = 0.1084, *p* = 0.7419) or in the MLA group (*X2* (1, *N* = 238) = 0.0239, *p* = 0.8771).

**TABLE A.1:** Experimental variables.

Name	Description	Operationalize
Independent variables (design)		
Source	Recommendation made by a machine learning algorithm or human analyst	Binary (A)
Consistency	Recommendation that is consistent with the report or not consistent	Binary (A)
Membership to group	Membership to either the SecA or the MLA group	Binary (B)
Assignment to specific order	Random assignments of participants to an order in which they will go through the manipulations.	Nominal (C)
*Background variables*		
Experience in performing vulnerability risk assessments	Self‐reported experience in performing vulnerability risk assessments	Ordinal scale (D)
Experience in using machine learning algorithms	Self‐reported experience in using machine learning algorithms	Ordinal scale (D)
Experience in developing machine learning algorithms	Self‐reported experience in developing machine learning algorithms	Ordinal scale (D)
Experience in reading threat intelligence reports	Self‐reported experience in reading threat intelligence reports	Ordinal scale (D)
*Dependent variables*		
Agreement with recommendation	Item on whether the participant agrees with the recommendation	Binary (E)
Perception of bias of the recommendation	Self‐reported perception of the bias of the recommendation	Ordinal scale (F)
Perception of bias of the report	Self‐reported perception of the bias of the report	Ordinal scale (F)
Perception of bias of information	Aggregated perception of the bias of information	Ordinal scale (J)
Perception of usability recommendation source	Self‐reported perception of the usability of a source	Ordinal scale (G)
Perception of quality recommendation source	Self‐reported perception of the quality of a source	Ordinal scale (G)
Perception of recommendation source	Aggregated Likert scale variable constituted of the perception of usability and the quality	Ordinal scale (J)
Difference score source	Aggregated Likert scale variable constituted of the perception of usability and the quality	Ordinal scale (K)
*Assessment validation variables*		
Manipulation check	Check question on who made the internal recommendation	Binary (H)
Attention check	Two questions on reported information	Nominal (See 13)
Likelihood of exploitation assessment	Categorical assessment of the likelihood	Ordinal (I)
Explanation answers	Explanations on why the specific answer is chosen	Open Text
*Experiment validation variables*		
Task understanding	Self‐reported perception of the participants that they had a clear understanding of the task	Ordinal scale (F)
Task time	Self‐reported perception of the participants that they had enough time to complete the task	Ordinal scale (F)
Task training	Self‐reported perception of the participants that the training material was sufficient to carry out the tasks	Ordinal scale (F)
Task material	Suggestions that can be useful to complete the tasks	Open Text

*Note*: (A) Based on randomization order and according to balanced design.

*Note*: (B) Based on when and where the measurement took place.

*Note*: (C) Automatically performed by the Qualtrics submission tool.

*Note*: (D) Multiple choice: no experience, as part of university projects, one or two times outside of university, several times outside of university/analyzing reports is part of job outside of university.

*Note*: (E) Multiple choice: Yes or no.

*Note*: (F) Five‐point Likert scale: strongly disagree, disagree, neither‐agree‐nor‐disagree/neutral, agree, strongly agree.

*Note*: (G) Five‐point Likert scale: very poor, poor, acceptable, good, very good.

*Note*: (H) Multiple choice: *HackHunter, the machine learning algorithm* or *A. Milliband, the human analyst*.

*Note*: (I) Multiple choice: low, medium, high.

*Note*: (J) Score between 1 and 5.

**TABLE A.2:** Attention checks included.

Report ID	Report	Attention check	Possible answers
1	NCSC‐2023‐0428	What is required for successful exploitation? Choose the reported option.	**Access to the management interface**
			Authentication
			User interaction
		Finish the sentence: According to Ivanti, the vulnerability has…	Never been exploited
			Been exploited in a random manner
			**Been exploited in a targeted manner**
2	NCSC‐2023‐0277	What can the attacker do after successful exploitation? Choose the reported option.	**Execute arbitrary code**
			Escalate privileges
			Install a backdoor
		Finish the sentence: The malicious party…	Does need full prior authentication for this
			**Does not need prior authentication for this**
			Needs some prior authentication for this
3	NCSC‐2023‐0282	Successful exploitation could lead an attacker to? Choose the reported option	Gain access to the management interface
			**Potentially take over the system**
			Install a backdoor
		Finish the sentence: According to Fortinet, there is an actor…	Who abuses only newer vulnerabilities
			Who abused older, but dissimilar vulnerabilities
			**Who abused older, similar vulnerabilities**
4	NCSC‐2022‐0368	What is required for successful exploitation? Choose the reported option.	**Access to the management environment**
			Authentication
			User interaction
		Finish the sentence: According to the NCSC, Security researchers have reported that the vulnerabilities…	Are not being actively exploited
			**Are being actively exploited in combination with another vulnerability**
			Are being actively exploited separately
5	NCSC‐2022‐0334	What is required for successful exploitation? Choose the reported option.	**Have access to the self‐IP or management interface of the BIG‐IP system**
			Have access to users of the BIG‐IP system
			User interaction
		Finish the sentence: According to the NCSC, the research method is…	Complicated
			**Quite simple**
			Medium difficulty
6	NCSC‐2022‐0056	What is required for successful exploitation? Choose the reported option.	Access to the management interface
			**SAML SSO authentication must be enabled**
			User interaction
		Finish the sentence: OpenSUSE has released updates to fix the vulnerabilities in…	Leap 10
			**Leap 15.3**
			Leap 11.3
7	NCSC‐2023‐0256	Successful exploitation could lead an attacker to? Choose the reported option.	Install a backdoor
			Escalate privileges
			**Gain access to the vulnerable system**
		Finish the sentence: Barracuda Networks…	Has not published Indicators of Compromise
			**Has published Indicators of Compromise**
			Has not found Indicators of Compromise
8	NCSC‐2023‐0346	Successful exploitation could lead an attacker to? Choose the reported option.	Access the management interface
			**Bypass a security measure**
			Install a backdoor
		Finish the sentence: It cannot be ruled out…	That the attacker can become domain administrator
			That the attacker could install multiple backdoors
			**That the attacker can gain access to sensitive data**

*Note*: Table with the attention checks and possible answers per report. The correct answer has been made **bold**.

In addition, the difference in reports was analyzed. Real reports were abbreviated, and there are inherent differences between these reports. For example, there is more background information available in one report than in another. Since every participant went through a certain condition twice with two different reports (i.e., scenarios), the theoretical analysis for these type of results requires that these scenarios need to be sufficiently different to avoid a learning effect (Massacci et al. 2024). Here, the learning effect was analyzed by looking at the dependent variable of overall perception of bias. The expectation is that there is no difference in outcome between each pair of reports, see Table 3 for the pairs of reports (e.g., 1 and 8 are in both groups paired). Wilcoxon signed‐rank tests were performed to see whether these scores represented a difference in repeated measures. As expected from Massacci et al. (2024), no significant results were obtained. See Table A.3 for the results. Thus, we conclude that there was no major learning effect occurring in our data.

**TABLE A.3:** Results learning effect: Wilcoxon signed‐rank test.

Condition	Median–mean report	Median–mean paired report	T	*p*
Security aware group (SecA) (*N* = 218)				
H‐C	2.50–2.53	2.50–2.41	43.5	0.5710
H‐I	2.50–2.42	2.50–2.61	55.0	0.3064
A‐C	2.00–2.10	2.50–2.57	50.5	0.1246
A‐I	2.50–2.59	3.00–2.86	62.5	0.3150
**Machine learning aware group (MLA) (** *N* **= 238)**				
H‐C	2.75–2.51	3.00–2.80	81.0	0.2291
H‐I	2.50–2.68	3.00–2.77	92.5	0.6400
A‐C	3.00–3.08	3.00–2.94	55.0	0.4994
A‐I	3.00–3.00	2.50–2.70	75.0	0.1568
Aggregated group				
H‐C	2.50–2.53	2.50–2.51	304.0	0.8631
H‐I	2.50–2.56	2.63–2.69	352.5	0.5945
A‐C	2.50–2.62	2.75–2.76	244.0	0.5131
A‐I	2.75–2.81	2.88–2.78	313.0	0.9738

*Note*: H‐C Recommendation given by human and consistent with the report.

*Note*: H‐I Recommendation given by human and inconsistent with the report.

*Note*: A‐C Recommendation given by algorithm and consistent with the report.

*Note*: A‐I Recommendation given by algorithm and inconsistent with the report.

## Report 3: All Conditions

Figures B.1 and B.2 show the third abbreviated report for all conditions. It can be observed that the underlying source of the final internal recommendation is either human (A. Milliband) or algorithmic (HackHunter). Gauging whether the recommendation is consistent can be based upon the additional pieces of threat intelligence.

## References

[risa70178-bib-0001] Aghakhani, N. , O. Oh , D. Gregg , and H. Jain . 2022. “How Review Quality and Source Credibility Interacts to Affect Review Usefulness: An Expansion of the Elaboration Likelihood Model.” Information Systems Frontiers 25: 1–19.

[risa70178-bib-0002] Allodi, L. , M. Cremoninih , F. Massacci , and W. Shim . 2020. “Measuring the Accuracy of Software Vulnerability Assessments: Experiments With Students and Professionals.” 25, no. 2: 1063–1094.

[risa70178-bib-0003] Allodi, L. , and F. Massacci . 2014. “Comparing Vulnerability Severity and Exploits Using Case‐Control Studies.” ACM Transactions on Information and System Security (TISSEC) 17, no. 1: 1–20.

[risa70178-bib-0004] Allodi, L. , and F. Massacci . 2017. “Security Events and Vulnerability Data for Cybersecurity Risk Estimation.” Risk Analysis 37, no. 8: 1606–1627. 28800378 10.1111/risa.12864

[risa70178-bib-0005] Balog‐Way, D. , K. McComas , and J. Besley . 2020. “The Evolving Field of Risk Communication.” Risk Analysis 40, no. S1: 2240–2262. 33084114 10.1111/risa.13615 PMC7756860

[risa70178-bib-0006] Basyurt, A. S. , J. Fromm , S. Stieglitz , and M. Mirbabaie . 2022. “Credibility of Cyber Threat Communication on Twitter–Expert Evaluation of Indicators for Automated Credibility Assessment.”*Wirtschaftsinformatik 2022 Proceedings*. 2.

[risa70178-bib-0007] Bellprat, O. , V. Guemas , F. Doblas‐Reyes , and M. G. Donat . 2019. “Towards Reliable Extreme Weather and Climate Event Attribution.” Nature Communications 10, no. 1: 1732. 10.1038/s41467-019-09729-2 PMC6465259 30988387

[risa70178-bib-0008] Belton, B. K. , and M. K. Dhami . 2020. Cognitive Biases and Debiasing in Intelligence Analysis Routledge.

[risa70178-bib-0009] Biden, J. R. J. 2021. Executive Order on Improving the Nation's Cybersecurity. White House.

[risa70178-bib-0010] Birnbaum, M. H. , and S. E. Stegner . 1979. “Source Credibility in Social Judgment: Bias, Expertise, and the Judge's Point of View.” Journal of Personality and Social Psychology 37, no. 1: 48–74.

[risa70178-bib-0011] Bouwman, X. , H. Griffioen , J. Egbers , C. Doerr , B. Klievink , and M. Van Eeten . 2020. “A Different Cup of { TI }? the Added Value of Commercial Threat Intelligence.” In *29th USENIX Security Symposium (USENIX Security 20)*, 433–450.

[risa70178-bib-0012] Bozorgi, M. , L. K. Saul , S. Savage , and G. M. Voelker . 2010. “Beyond Heuristics: Learning to Classify Vulnerabilities and Predict Exploits.” In *Proceedings of the 16th ACM SIGKDD International Conference on Knowledge Discovery and Data Mining*, 105–114.

[risa70178-bib-0013] Burton, J. W. , M.‐K. Stein , and T. B. Jensen . 2020. “A Systematic Review of Algorithm Aversion in Augmented Decision Making.” Journal of Behavioral Decision Making 33, no. 2: 220–239.

[risa70178-bib-0014] Caltagirone, S. 2014. 15 Things Wrong With Today's Threat Intelligence Reporting. https://www.activeresponse.org/15‐things‐wrong‐with‐todays‐threat‐intelligence‐reporting/.

[risa70178-bib-0015] Castelo, N. , M. Bos , and D. R. Lehmann . 2019. “Task‐Dependent Algorithm Aversion.” Journal of Marketing Research 56, no. 5: 809–825.

[risa70178-bib-0016] Chong, C. Y. , P. Thongtanunam , and C. Tantithamthavorn . 2021. “Assessing the Students' Understanding and Their Mistakes in Code Review Checklists: An Experience Report of 1,791 Code Review Checklist Questions From 394 Students.”*IEEE*, 20–29.

[risa70178-bib-0017] Chugunova, M. , and D. Sele . 2022. “We and It: An Interdisciplinary Review of the Experimental Evidence on How Humans Interact With Machines.” Journal of Behavioral and Experimental Economics 99: 101897.

[risa70178-bib-0018] Chun, K. J. , and H. K. Park . 1998. “Examining the Conflicting Results of GDSS Research.” Information & Management 33, no. 6: 313–325.

[risa70178-bib-0019] Collier, Z. A. , R. J. Gruss , and A. S. Abrahams . 2023. “How Good Are Large Language Models at Product Risk Assessment?” Risk Analysis 43, no. 3: 590–604. 38851858 10.1111/risa.14351 PMC12032383

[risa70178-bib-0020] Committee, C. T. I. T. 2023. Introduction to STIX. https://oasis‐open.github.io/cti‐documentation/stix/intro.html.

[risa70178-bib-0021] Commons, M. L. , P. M. Miller , and T. G. Gutheil . 2004. “Expert Witness Perceptions of Bias in Experts.” Journal of the American Academy of Psychiatry and the Law Online 32, no. 1: 70–75. 15497632

[risa70178-bib-0022] Commons, M. L. , P. M. Miller , E. Y. Li , and T. G. Gutheil . 2012. “Forensic Experts' Perceptions of Expert Bias.” International Journal of Law and Psychiatry 35, no. 5–6: 362–371. 23046867 10.1016/j.ijlp.2012.09.016

[risa70178-bib-0023] Corporation, T. M. 2023. MITRE ATT&CK. https://attack.mitre.org/.

[risa70178-bib-0024] Cox, T. 2008. “What's Wrong With Risk Matrices?” Risk Analysis 28, no. 2: 497–512. 18419665 10.1111/j.1539-6924.2008.01030.x

[risa70178-bib-0025] de Smale, S. , R. van Dijk , X. Bouwman , J. van der Ham , and M. van Eeten . 2023. “No One Drinks From the Firehose: How Organizations Filter and Prioritize Vulnerability Information.” In 2023 IEEE Symposium on Security and Privacy (SP). IEEE.

[risa70178-bib-0026] Di Tizio, G. , M. Armellini , and F. Massacci . 2022. “Software Updates Strategies: A Quantitative Evaluation Against Advanced Persistent Threats.” IEEE Transactions on Software Engineering 49, no. 3: 1359–1373.

[risa70178-bib-0027] Dietvorst, B. J. , and S. Bharti . 2020. “People Reject Algorithms in Uncertain Decision Domains Because They Have Diminishing Sensitivity to Forecasting Error.” Psychological Science 31, no. 10: 1302–1314. 32916083 10.1177/0956797620948841

[risa70178-bib-0028] Dietvorst, B. J. , J. P. Simmons , and C. Massey . 2015. “Algorithm Aversion: People Erroneously Avoid Algorithms After Seeing Them Err.” Journal of Experimental Psychology: General 144, no. 1: 114–126. 25401381 10.1037/xge0000033

[risa70178-bib-0029] Dubois, D. 2010. “Representation, Propagation, and Decision Issues in Risk Analysis Under Incomplete Probabilistic Information.” Risk Analysis: An International Journal 30, no. 3: 361–368. 10.1111/j.1539-6924.2010.01359.x 20487395

[risa70178-bib-0030] Durbach, I. N. , and T. J. Stewart . 2011. “An Experimental Study of the Effect of Uncertainty Representation on Decision Making.” European Journal of Operational Research 214, no. 2: 380–392.

[risa70178-bib-0031] Dzindolet, M. T. , L. G. Pierce , H. P. Beck , and L. A. Dawe . 2002. “The Perceived Utility of Human and Automated Aids in a Visual Detection Task.” Human Factors 44: 79–97. 12118875 10.1518/0018720024494856

[risa70178-bib-0032] Egloff, F. J. 2020. “Contested Public Attributions of Cyber Incidents and the Role of Academia.” Contemporary Security Policy 41, no. 1: 55–81.

[risa70178-bib-0033] Feng, X. , and J. Gao . 2020. “Is Optimal Recommendation the Best? A Laboratory Investigation Under the Newsvendor Problem.” Decision Support Systems 131: 113251.

[risa70178-bib-0034] Finucane, M. L. , A. Alhakami , P. Slovic , and S. M. Johnson . 2000. “The Affect Heuristic in Judgments of Risks and Benefits.” Journal of Behavioral Decision Making 13, no. 1: 1–17.

[risa70178-bib-0035] Fischhoff, B. 1982. “Debiasing.” In Judgment Under Uncertainty: Heuristics and Biases, edited by D. Kahneman , P. Slovic , and A. Tversky , 422–444. Cambridge University Press. 10.1126/science.185.4157.1124 17835457

[risa70178-bib-0036] Flanagin, A. J. , and M. J. Metzger . 2020. Source Credibility. John Wiley & Sons, Ltd.

[risa70178-bib-0037] Florig, H. K. , M. G. Morgan , K. M. Morgan , et al. 2001. “A Deliberative Method for Ranking Risks (i): Overview and Test Bed Development.” Risk Analysis 21, no. 5: 913–913. 11798126 10.1111/0272-4332.215161

[risa70178-bib-0038] Food and Drug Administration . 2001. Guidance for Industry: Statistical Approaches to Establishing Bioequivalence. Food and Drug Administration.

[risa70178-bib-0039] Giest, S. , and A. Samuels . 2023. “Administrative Burden in Digital Public Service Delivery: The Social Infrastructure of Library Programs for E‐Inclusion.” Review of Policy Research 40, no. 5: 626–645.

[risa70178-bib-0040] Gigerenzer, G. , and W. Gaissmaier . 2011. “Heuristic Decision Making.” Annual Review of Psychology 62: 451–482. 10.1146/annurev-psych-120709-145346 21126183

[risa70178-bib-0041] Gill, H. , E. Vreeker‐Williamson , L. S. Hing , S. A. Cassidy , and K. Boies . 2024. “Effects of Cognition‐Based and Affect‐Based Trust Attitudes on Trust Intentions.” Journal of Business and Psychology 39, no. 6: 1355–1374. 39507389 10.1007/s10869-024-09986-z PMC11534891

[risa70178-bib-0042] Gönül, M. S. , D. Önkal , and M. Lawrence . 2006. “The Effects of Structural Characteristics of Explanations on Use of a DSS.” Decision Support Systems 42, no. 3: 1481–1493.

[risa70178-bib-0043] Goddard, K. , A. Roudsari , and J. C. Wyatt . 2012. “Automation Bias: A Systematic Review of Frequency, Effect Mediators, and Mitigators.” Journal of the American Medical Informatics Association 19, no. 1: 121–127. 21685142 10.1136/amiajnl-2011-000089 PMC3240751

[risa70178-bib-0044] Hausken, K. , J. W. Welburn , and J. Zhuang . 2024. “A Review of Attacker–Defender Games and Cyber Security.” Games 15, no. 4: 28.

[risa70178-bib-0045] Hou, Y. T.‐Y. , and M. F. Jung . 2021. “Who Is the Expert? Reconciling Algorithm Aversion and Algorithm Appreciation in AI‐Supported Decision Making.” Proceedings of the ACM on Human‐Computer Interaction 5.

[risa70178-bib-0046] Hovland, C. I. , and W. Weiss . 1951. “The Influence of Source Credibility on Communication Effectiveness.” Public Opinion Quarterly 15, no. 4: 635–650.

[risa70178-bib-0047] Initiative, N. J. T. F. T. 2012. NIST SP 800‐30 Rev. 1: Guide for Conducting Risk Assessments. https://csrc.nist.gov/pubs/sp/800/30/r1/final.

[risa70178-bib-0048] Irshad, E. , and A. Basit Siddiqui . 2023. “Cyber Threat Attribution Using Unstructured Reports in Cyber Threat Intelligence.” Egyptian Informatics Journal 24, no. 1: 43–59.

[risa70178-bib-0049] Irwin, D. , and D. R. Mandel . 2019. “Improving Information Evaluation for Intelligence Production.” Intelligence and National Security 34, no. 4: 503–525.

[risa70178-bib-0050] Irwin, D. , and D. R. Mandel . 2023. “Communicating Uncertainty in National Security Intelligence: Expert and Nonexpert Interpretations of and Preferences for Verbal and Numeric Formats.” Risk Analysis 43, no. 5: 943–957. 35994518 10.1111/risa.14009

[risa70178-bib-0051] Isaksen, B. G. M. , and K. R. McNaught . 2019. “Uncertainty Handling in Estimative Intelligence—Challenges and Requirements From Both Analyst and Consumer Perspectives.” Journal of Risk Research 22, no. 5: 643–657.

[risa70178-bib-0052] Ismagilova, E. , E. Slade , N. P. Rana , and Y. K. Dwivedi . 2020. “The Effect of Characteristics of Source Credibility on Consumer Behaviour: A Meta‐Analysis.” Journal of Retailing and Consumer Services 53: 101736.

[risa70178-bib-0053] Jacobs, J. , S. Romanosky , O. Suciu , B. Edwards , and A. Sarabi . 2023. “Enhancing Vulnerability Prioritization: Data‐Driven Exploit Predictions With Community‐Driven Insights.” In 2023 IEEE European Symposium on Security and Privacy Workshops (EuroS&PW), 194–206. IEEE.

[risa70178-bib-0054] Jaspersen, J. G. , and G. Montibeller . 2015. “Probability Elicitation Under Severe Time Pressure: A Rank‐Based Method.” Risk Analysis 35, no. 7: 1317–1335. 25850859 10.1111/risa.12357

[risa70178-bib-0055] Jensen, M. A. 2012. “Intelligence Failures: What Are They Really and What Do We Do About Them?” Intelligence and National Security 27, no. 2: 261–282.

[risa70178-bib-0056] Johnson, C. , L. Badger , D. Waltermire , J. Snyder , and C. Skorupka . 2012. NIST Special Publication 800‐150: Guide to Cyber Threat Information Sharing. 10.6028/NIST.SP.800-150.

[risa70178-bib-0057] Kahneman, D. , and G. Klein . 2009. “Conditions for Intuitive Expertise: A Failure To Disagree.” American Psychologist 64, no. 6: 515–526. 19739881 10.1037/a0016755

[risa70178-bib-0058] Kahneman, D. , and A. Tversky . 1979. “Prospect Theory: An Analysis of Decision Under Risk.” Econometrica 47, no. 2: 263–291.

[risa70178-bib-0059] Kamara, I. , R. Leenes , K. Stuurman , and V. J. Boom . 2020. “The Cybersecurity Certification Landscape in the Netherlands After the Union Cybersecurity Act.”*Tilburg Institute for Law, Technology, and Society* .

[risa70178-bib-0060] Karvetski, C. W. , D. R. Mandel , and D. Irwin . 2020. “Improving Probability Judgment in Intelligence Analysis: From Structured Analysis to Statistical Aggregation.” Risk Analysis 40, no. 5: 1040–1057. 32065440 10.1111/risa.13443

[risa70178-bib-0061] Kasperson, R. E. , T. Webler , B. Ram , and J. Sutton . 2022. “The Social Amplification of Risk Framework: New Perspectives.” Risk Analysis 42, no. 7: 1367–1380. 35861634 10.1111/risa.13926 PMC10360138

[risa70178-bib-0062] Kaufmann, E. , A. Chacon , E. E. Kausel , N. Herrera , and T. Reyes . 2023. “Task‐Specific Algorithm Advice Acceptance: A Review and Directions for Future Research.” Data and Information Management 7, no. 3: 100040.

[risa70178-bib-0063] Kawaguchi, K. 2021. “When Will Workers Follow an Algorithm? A Field Experiment With a Retail Business.” Management Science 67, no. 3: 1670–1695.

[risa70178-bib-0064] Kelly, S. , S. Kaye , and O. Oviedo‐Trespalacios . 2023. “What Factors Contribute to the Acceptance of Artificial Intelligence? A Systematic Review.” Telematics Informatics 77: 101925.

[risa70178-bib-0065] Khan, S. , and S. Parkinson . 2018. Review Into State of the Art of Vulnerability Assessment Using Artificial Intelligence. Springer International Publishing.

[risa70178-bib-0066] Klein, G. , M. Jalaeian , R. R. Hoffman , and S. T. Mueller . 2023. “The Plausibility Transition Model for Sensemaking.” Frontiers in Psychology 14: 1160132. 37303907 10.3389/fpsyg.2023.1160132 PMC10251660

[risa70178-bib-0067] Klein, G. , J. K. Phillips , E. L. Rall , and D. A. Peluso . 2007. A Data/Frame Theory of Sensemaking. Taylor and Francis.

[risa70178-bib-0068] Labunets, K. , F. Massacci , F. Paci , and K. Tuma . 2023. “A New, Evidence‐Based, Theory for Knowledge Reuse in Security Risk Analysis.” Empirical Software Engineering 28, no. 4: 90.

[risa70178-bib-0069] Landon‐Murray, M. 2016. “Big Data and Intelligence: Applications, Human Capital, and Education.” Journal of Strategic Security 9: 94–123.

[risa70178-bib-0070] Loewenstein, G. F. , E. U. Weber , C. K. Hsee , and N. Welch . 2001. “Risk as Feelings.” Psychological Bulletin 127, no. 2: 267–286. 11316014 10.1037/0033-2909.127.2.267

[risa70178-bib-0071] Logg, J. M. , J. A. Minson , and D. A. Moore . 2019. “Algorithm Appreciation: People Prefer Algorithmic to Human Judgment.” Organizational Behavior and Human Decision Processes 151: 90–103.

[risa70178-bib-0072] Mahmud, H. , A. Najmul Islam , S. I. Ahmed , and K. Smolander . 2022. “What Influences Algorithmic Decision‐Making? A Systematic Literature Review on Algorithm Aversion.” Technological Forecasting and Social Change 175: 121390.

[risa70178-bib-0073] Makridis, C. , L. Maschmeyer , and M. Smeets . 2024. “If It Bleeps It Leads? Media Coverage on Cyber Conflict and Misperception.” Journal of Peace Research 61, no. 1: 72–86.

[risa70178-bib-0074] Mandel, D. R. 2020. “Assessment and Communication of Uncertainty in Intelligence to Support Decision‐Making.” NATO STO TECHNICAL REPORT, TR‐SAS‐114.

[risa70178-bib-0075] Mandel, D. R. , and A. Barnes . 2014. “Accuracy of Strategic Intelligence Forecasts.” National Academy of Sciences 11: 10984–10989. 10.1073/pnas.1406138111 PMC4121776 25024176

[risa70178-bib-0076] Mandel, D. R. , D. Irwin , M. K. Dhami , and D. V. Budescu . 2023. “Meta‐informational Cue Inconsistency and Judgment of Information Accuracy: Spotlight on Intelligence Analysis.” Journal of Behavioral Decision Making 36, no. 3: e2307.

[risa70178-bib-0077] Maschmeyer, L. , R. J. Deibert , and J. R. Lindsay . 2021. “A Tale of Two Cybers—How Threat Reporting by Cybersecurity Firms Systematically Underrepresents Threats to Civil Society.” Journal of Information Technology & Politics 18, no. 1: 1–20.

[risa70178-bib-0078] Massacci, F. , A. Papotti , and R. Paramitha . 2024. “Addressing Combinatorial Experiments and Scarcity of Subjects by Provably Orthogonal and Crossover Experimental Designs.” *Journal of Systems and Software* 211: 1–18. 111990.

[risa70178-bib-0079] Menkveld, C. 2020. “Understanding the Complexity of Intelligence Problems.” Intelligence and National Security 36, no. 5: 621–641.

[risa70178-bib-0080] Meyners, M. 2012. “Equivalence Tests—A Review.” Food Quality and Preference 26, no. 2: 231–245.

[risa70178-bib-0081] Morgan, K. M. , M. L. DeKay , P. S. Fischbeck , M. G. Morgan , B. Fischhoff , and H. K. Florig . 2001. “A Deliberative Method for Ranking Risks (ii): Evaluation of Validity and Agreement Among Risk Managers.” Risk Analysis 21, no. 5: 923–923. 11798127 10.1111/0272-4332.215162

[risa70178-bib-0082] Naiakshina, A. , A. Danilova , C. Tiefenau , M. Herzog , S. Dechand , and M. Smith . 2017. “Why Do Developers Get Password Storage Wrong? A Qualitative Usability Study.” In *Proceedings of the 24th ACM SIGSAC Conference on Computer and Communications Security* (*ccs'*17), 311–328.

[risa70178-bib-0083] Naiakshina, A. , A. Danilova , C. Tiefenau , and M. Smith . 2018. “Deception Task Design in Developer Password Studies: Exploring A Student Sample.” In *Proceedings of the 14th USENIX Symposium on Usable Privacy and Security (soups'18)*, 297–313.

[risa70178-bib-0084] NCSC . 2023a. About Us. https://www.ncsc.nl/over‐ncsc.

[risa70178-bib-0085] NCSC . 2023b. Inschalingsmatrix. https://www.ncsc.nl/documenten/publicaties/2019/juli/02/inschalingsmatrix.

[risa70178-bib-0086] NCSC . 2023c. What Is a NCSC Advisory. https://www.ncsc.nl/documenten/publicaties/2019/juli/02/wat‐is‐een‐ncsc‐beveiligingsadvies.

[risa70178-bib-0087] Okoli, O. , G. Weller , and J. Watt . 2016. “Information Processing and Intuitive Decision‐Making on the Fireground: Towards a Model of Expert Intuition.” Cognition, Technology & Work 18: 89–103.

[risa70178-bib-0088] Ormond, D. , M. Warkentin , A. C. Johnston , and S. C. Thompson . 2016. “Perceived Deception: Evaluating Source Credibility and Self‐Efficacy.” Journal of Information Privacy and Security 12, no. 4: 197–217.

[risa70178-bib-0089] Otway, H. , and D. von Winterfeldt . 1992. “Expert Judgment in Risk Analysis and Management: Process, Context, and Pitfalls.” Risk Analysis 12, no. 1: 83–93. 1574619 10.1111/j.1539-6924.1992.tb01310.x

[risa70178-bib-0090] Paté‐Cornell, E. 2002. “Fusion of Intelligence Information: A Bayesian Approach.” Risk Analysis 22, no. 3: 445–454. 12088224 10.1111/0272-4332.00056

[risa70178-bib-0091] Paté‐Cornell, E. 2024. “Preferences in AI Algorithms: The Need for Relevant Risk Attitudes in Automated Decisions Under Uncertainties.”*Risk Analysis* . 10.1111/risa.14268 38184297

[risa70178-bib-0092] Pedersen, T. , and P. T. Jansen . 2019. “Seduced By Secrecy—Perplexed by Complexity: Effects of Secret vs Open‐Source on Intelligence Credibility and Analytic Confidence.” Intelligence and National Security 34, no. 6: 881–898.

[risa70178-bib-0093] Perry, L. , B. Shapira , and R. Puzis . 2019. “NO‐DOUBT: Attack Attribution Based on Threat Intelligence Reports.” In *2019 IEEE International Conference on Intelligence and Security Informatics (ISI)*, 80–85.

[risa70178-bib-0094] Prahl, A. , and L. Van Swol . 2017. “Understanding Algorithm Aversion: When Is Advice From Automation Discounted?” Journal of Forecasting 36, no. 6: 691–702.

[risa70178-bib-0095] Ranade, P. , A. Piplai , S. Mittal , A. Joshi , and T. Finin . 2021. “Generating Fake Cyber Threat Intelligence Using Transformer‐Based Models.” In 2021 International Joint Conference on Neural Networks (IJCNN), 1–9. IJCNN.

[risa70178-bib-0096] Rieger, T. , L. Kugler , D. Manzey , and E. Roesler . 2023. “The (Im)perfect Automation Schema: Who Is Trusted More, Automated or Human Decision Support?” Human Factors 66, no. 8: 1995–2007. 10.1177/00187208231197347. 37632728

[risa70178-bib-0097] Rong, G. , J. Li , M. Xie , and T. Zheng . 2012. “The Effect of Checklist in Code Review for Inexperienced Students: An Empirical Study.” In Proceedings of the 25th IEEE Conference on Software Engineering Education and Training (cseet'12), 120–124. IEEE.

[risa70178-bib-0098] Salman, I. , A. T. Misirli , and N. Juristo . 2015. “Are Students Representatives of Professionals in Software Engineering Experiments?” In Proceedings of the 37th IEEE/ACM International Conference on Software Engineering (icse'15), 666–676. IEEE.

[risa70178-bib-0099] Schaberreiter, T. , V. Kupfersberger , K. Rantos , et al. 2019. “A Quantitative Evaluation of Trust in the Quality of Cyber Threat Intelligence Sources.” In Proceedings of the 14th International Conference on Availability, Reliability and Security. ACM.

[risa70178-bib-0100] Scharrer, L. , M. Stadtler , and R. Bromme . 2019. “Biased Recipients Encounter Biased Sources: Effect of Ethical (Dis‐)Agreement Between Recipient and Author on Evaluating Scientific Claims.” Applied Cognitive Psychology 33, no. 6: 1165–1177.

[risa70178-bib-0101] Scholz, R. W. , and R. Hansmann . 2007. “Combining Experts' Risk Judgments on Technology Performance of Phytoremediation: Self‐Confidence Ratings, Averaging Procedures, and Formative Consensus Building.” Risk Analysis: An International Journal 27, no. 1: 225–240. 10.1111/j.1539-6924.2006.00871.x 17362411

[risa70178-bib-0102] Schuirmann, D. 1981. “On Hypothesis‐Testing to Determine if the Mean of a Normal‐Distribution Is Contained in a Known Interval.” Biometrics 37, no. 3: 617–617.

[risa70178-bib-0103] Selten, F. , and B. Klievink . 2024. “Organizing Public Sector AI Adoption: Navigating Between Separation and Integration.” Government Information Quarterly 41, no. 1: 101885.

[risa70178-bib-0104] Siegrist, M. 2021. “Trust and Risk Perception: A Critical Review of the Literature.” Risk Analysis 41, no. 3: 480–490. 31046144 10.1111/risa.13325

[risa70178-bib-0105] Skitka, L. J. , K. Mosier , and M. D. Burdick . 2000. “Accountability and Automation Bias.” International Journal of Human‐Computer Studies 52, no. 4: 701–717.

[risa70178-bib-0106] Slovic, P. 1966. “Cue‐Consistency and Cue‐Utilization in Judgment.” American Journal of Psychology 79, no. 3: 427–434. 5968479

[risa70178-bib-0107] Slovic, P. 1987. “Perception of Risk.” Science 236, no. 4799: 280–285. 3563507 10.1126/science.3563507

[risa70178-bib-0108] Slovic, P. , M. L. Finucane , E. Peters , and D. G. MacGregor . 2007. “The Affect Heuristic.” European Journal of Operational Research 177, no. 3: 1333–1352.

[risa70178-bib-0109] Sniezek, J. A. 1992. “Groups Under Uncertainty: An Examination of Confidence in Group Decision Making.” Organizational Behavior and Human Decision Processes 52, no. 1: 124–155.

[risa70178-bib-0110] Spiegelhalter, D. 2017. “Risk and Uncertainty Communication.” Annual Review of Statistics and Its Application 4: 31–60.

[risa70178-bib-0111] Stroop, J. R. 1932. “Is the Judgment of the Group Better Than That of the Average Member of the Group?” Journal of experimental Psychology 15, no. 5: 550.

[risa70178-bib-0112] Tahaei, M. , and K. Vaniea . 2022. “Recruiting Participants With Programming Skills: A Comparison of Four Crowdsourcing Platforms and a CS Student Mailing List.” In *Proceedings of the 2022 CHI Conference on Human Factors in Computing Systems* .

[risa70178-bib-0113] Tounsi, W. , and H. Rais . 2018. “A Survey on Technical Threat Intelligence in the Age of Sophisticated Cyber Attacks.” Computers & Security 72: 212–233.

[risa70178-bib-0114] Tsui, K.‐L. 1992. “An Overview of Taguchi Method and Newly Developed Statistical Methods for Robust Design.” IIE Transactions 24, no. 5: 44–57.

[risa70178-bib-0115] van Gerwen, S. , A. Papotti , K. Tuma , and F. Massacci . 2025. Algorithm Perception When Using Threat Intelligence in Vulnerability Risk Assessment—Replication Package. 10.5281/zenodo.16418848. PMC12857607 41615328

[risa70178-bib-0116] van Schaik, P. , K. Renaud , C. Wilson , J. Jansen , and J. Onibokun . 2020. “Risk as Affect: The Affect Heuristic in Cybersecurity.” Computers & Security 90: 101651.

[risa70178-bib-0117] Vielberth, M. , F. Böhm , I. Fichtinger , and G. Pernul . 2020. “Security Operations Center: A Systematic Study and Open Challenges.” IEEE Access 8: 227756–227779.

[risa70178-bib-0118] Von Walter, B. , D. Kremmel , and B. Jäger . 2022. “The Impact of Lay Beliefs About AI on Adoption of Algorithmic Advice.” Marketing Letters 33, no. 1: 143–155.

[risa70178-bib-0119] Wagner, T. D. , K. Mahbub , E. Palomar , and A. E. Abdallah . 2019. “Cyber Threat Intelligence Sharing: Survey and Research Directions.” Computers Security 87: 101589.

[risa70178-bib-0120] Wallace, L. E. , D. T. Wegener , and R. E. Petty . 2020. “When Sources Honestly Provide Their Biased Opinion: Bias as a Distinct Source Perception With Independent Effects on Credibility and Persuasion.” Personality and Social Psychology Bulletin 46, no. 3: 439–453. 31282841 10.1177/0146167219858654

[risa70178-bib-0121] Whitesmith, M. 2019. “The Efficacy of ACH in Mitigating Serial Position Effects and Confirmation Bias in an Intelligence Analysis Scenario.” Intelligence and National Security 34, no. 2: 225–242.

[risa70178-bib-0122] Whyte, C. E. 2022. “Machine Expertise in the Loop: Artificial Intelligence Decision‐Making Inputs and Cyber Conflict.” In 2022 14th International Conference on Cyber Conflict: Keep Moving! (CyCon), 135–154. IEEE.

[risa70178-bib-0123] Wiener, J. L. , and J. C. Mowen . 1986. “Source Credibility: On the Independent Effects of Trust and Expertise.” Advances in Consumer Research 13, no. 1: 306–310.

[risa70178-bib-0124] Winter, E. R. , V. Nowack , D. Bowes , et al. 2022. “Let's Talk With Developers, Not About Developers: A Review of Automatic Program Repair Research.” IEEE Transactions on Software Engineering, 419–436. IEEE.

[risa70178-bib-0125] Yang, W. , and K.‐Y. Lam . 2020. “Automated Cyber Threat Intelligence Reports Classification for Early Warning of Cyber Attacks in Next Generation SOC.” In Information and Communications Security, edited by J. Zhou , X. Luo , Q. Shen , and Z. Xu , 145–164. Springer International Publishing.

[risa70178-bib-0126] Zhang, Z. , H. Al Hamadi , E. Damiani , C. Y. Yeun , and F. Taher . 2022. “Explainable Artificial Intelligence Applications in Cyber Security: State‐of‐the‐Art in Research.” IEEE Access, 93104–93139. IEEE.

[risa70178-bib-0127] Zhang, Y. , and T. Liu . 2022. “Risk Assessment Based on a STPA–FMEA Method: A Case Study of a Sweeping Robot.” Risk Analysis 43, no. 3: 590–604. 35383984 10.1111/risa.13927

